# Metabolomics and transcriptomics combined with physiology reveal key metabolic pathway responses in tobacco roots exposed to NaHS

**DOI:** 10.1186/s12870-024-05402-z

**Published:** 2024-07-18

**Authors:** Wenjuan Yang, Dingxin Wen, Yong Yang, Hao Li, Chunlei Yang, Jun Yu, Haibo Xiang

**Affiliations:** 1 State Key Laboratory of Biocatalysis and Enzyme Engineering, School of Life Science, Hubei University, Wuhan, 430062 China; 2 Tobacco Research Institute of Hubei Province, Wuhan, 430030 China

**Keywords:** NaHS, Tobacco, Physiology, Transcriptome, Metabolome, Brassinosteroid, Aspartate

## Abstract

Hydrogen sulfide (H_2_S) has emerged as a novel endogenous gas signaling molecule, joining the ranks of nitric oxide (NO) and carbon monoxide (CO). Recent research has highlighted its involvement in various physiological processes, such as promoting root organogenesis, regulating stomatal movement and photosynthesis, and enhancing plant growth, development, and stress resistance. Tobacco, a significant cash crop crucial for farmers’ economic income, relies heavily on root development to affect leaf growth, disease resistance, chemical composition, and yield. Despite its importance, there remains a scarcity of studies investigating the role of H_2_S in promoting tobacco growth. This study exposed tobacco seedlings to different concentrations of NaHS (an exogenous H_2_S donor) − 0, 200, 400, 600, and 800 mg/L. Results indicated a positive correlation between NaHS concentration and root length, wet weight, root activity, and antioxidant enzymatic activities (CAT, SOD, and POD) in tobacco roots. Transcriptomic and metabolomic analyses revealed that treatment with 600 mg/L NaHS significantly effected 162 key genes, 44 key enzymes, and two metabolic pathways (brassinosteroid synthesis and aspartate biosynthesis) in tobacco seedlings. The addition of exogenous NaHS not only promoted tobacco root development but also potentially reduced pesticide usage, contributing to a more sustainable ecological environment. Overall, this study sheds light on the primary metabolic pathways involved in tobacco root response to NaHS, offering new genetic insights for future investigations into plant root development.

**Supplementary Information:**

The online version contains supplementary material available at 10.1186/s12870-024-05402-z.

## Introduction

Tobacco plants (*Nicotiana tabacum* L.) are a crucial crop in various countries and regions [1]. In order to maintain high productivity levels, a large quantity of pesticides is typically utilized to manage unwanted plants (weeds), insects, rodents, and fungi in tobacco fields [2]. This is especially prevalent during the seedling growth phase in greenhouses and when transplanting young plants into fields [3]. Pesticides may have both immediate acute effects in the short term and chronic effects over a longer period of time with repeated exposure to low concentrations. The excessive use of pesticides for tobacco disease control can result in soil, water, and air pollution, posing risks to human health, including tobacco farmers, and ecosystem well-being due to their toxicity, residues, and the development of pathogen resistance in organisms [4, 5]. Hence, reducing pesticide usage and adopting highly efficient and environmentally friendly formulations are imperative for the sustainable future of agriculture.

Hydrogen sulfide (H_2_S) is recognized as a crucial endogenous gasotransmitter that plays a key role in maintaining a dynamic balance in plants through various enzymes such as L-cysteine desulfhydrase (LCD), D-cysteine desulfhydrase (DCD), sulfite reductase, cyanoalanine synthase, cysteine synthase, and O-acetyl-l-serine(thiol)lyase [6]. While high concentrations of H_2_S can be malodorous and detrimental to plant growth, optimal levels serve as signaling molecules that regulate plant development and response to environmental stress [7]. In agricultural settings, the careful application of optimal H_2_S concentrations is involved in modulating several physiological processes essential for plant growth and development, including seed germination [8, 9], organogenesis [10, 11], adventitious and lateral root formation [12, 13], maturation, flower senescence [14], and enhanced tolerance to environmental stress [7, 15]. The role of H_2_S as a signaling molecule in orchestrating physiological processes in plants has garnered significant attention recently. These processes entail intricate interactions with hormones, reactive oxygen species (ROS), and other molecular signals [16]. For instance, Zou et al. demonstrated that pretreatment with an exogenous H_2_S donor (sodium hydrogen sulfide [NaHS]) elevated endogenous H_2_S levels, stimulated nodulation and nitrogen fixation activity, and enhanced nitrogen metabolism in soybean roots and nodules [17]. Furthermore, foliar application of NaHS led to a significant increase in flavin monooxygenase (FMO) activity and the expression of FMO-like proteins (*YUCCA2*), resulting in elevated levels of endogenous indole-3-acetic acid (IAA) and improved chilling tolerance in cucumber seedlings [16]. Treatment with NaHS also facilitated adventitious root formation by modulating osmotic substance levels and enhancing antioxidant capacity in cucumber plants [18]. The judicious use of NaHS may have the potential to reduce the necessity for frequent pesticide applications and enhance plant immunity.

Roots play a crucial role in providing crops with essential elements such as water, nutrients, hormones, and mechanical support for anchoring, ultimately effecting economic yield [19]. Despite typically representing only 10–20% of a plant’s total weight, a well-developed root is vital for healthy plant growth and development [6, 20]. Tobacco, a widely cultivated annual model crop and cash crop, relies heavily on root development for leaf growth, disease resistance, chemical composition, and overall yield [21]. While previous research has primarily focused on physiological processes, there remains a significant gap in understanding the broader changes in roots, malondialdehyde (MDA) accumulation, and enzymatic activities of catalase (CAT), superoxide dismutase (SOD), and peroxidase (POD), as well as key signaling pathways following H_2_S treatment in tobacco growth. Moreover, the underlying physiological, biochemical, and molecular mechanisms of how H_2_S regulates tobacco tolerance are still not fully understood, necessitating further comprehensive analysis and exploration.

Novel strategies are required to investigate the molecular mechanism by which NaHS enhances tobacco root development. Omics technology presents a unique opportunity to investigate the intricate regulatory networks involved in growth promotion [22]. Transcriptome sequencing technology, facilitated by the rapid advancements in sequencing tools, has become a popular method for deciphering gene function through comprehensive analysis of genomic data [23]. This approach offers a robust platform for systematically investigating the interplay between genotype and phenotype in transcriptional regulation. Transcriptome sequencing has been extensively employed in studying tobacco disease resistance and growth promotion, enabling the identification of differentially expressed genes across various biological processes. Nonetheless, challenges exist in distinguishing low abundance and unknown transcripts [23]. Liquid chromatography/mass spectrometry (LC/MS) is utilized to enhance metabolomics by detecting a wide array of compounds, surpassing traditional chemical analysis methods [24]. Metabolomics, a concept introduced by Nicholson et al., is considered a new omics field following transcriptomics, genomics, and proteomics research [25]. The integration of metabolome and transcriptome analysis has become a prevalent approach to investigate the regulatory networks and relationships between genes and metabolites [23, 26–30].

Limited research has been conducted on the transcriptional and metabolic mechanisms of NaHS in promoting tobacco root development. This study aimed to investigate the effect of NaHS pretreatment on tobacco seedlings by conducting physiological, transcriptomic, and metabolomic analyses on *Nicotiana tabacum* cultivar Yunyan 87. Utilizing targeted metabolomic technology, the study uncovered metabolic alterations induced by 600 mg/L NaHS. The results indicate that candidate genes and metabolites associated with the brassinosteroid synthetic pathway and aspartate metabolic pathway may play a role in regulating the development of tobacco seedlings. Furthermore, it was observed that a metabolic regulatory network was established in response to NaHS pretreatment. This research contributes to a deeper comprehension of the molecular mechanisms underlying the effects of NaHS on tobacco root development, offering a theoretical foundation for enhancing tobacco seedling production and developing NaHS-related products to promote tobacco growth, reduce pesticide usage, and safeguard the soil environment.

## Results

### Effects of exogenous NaHS on tobacco seedling growth

**Fig. 1:**
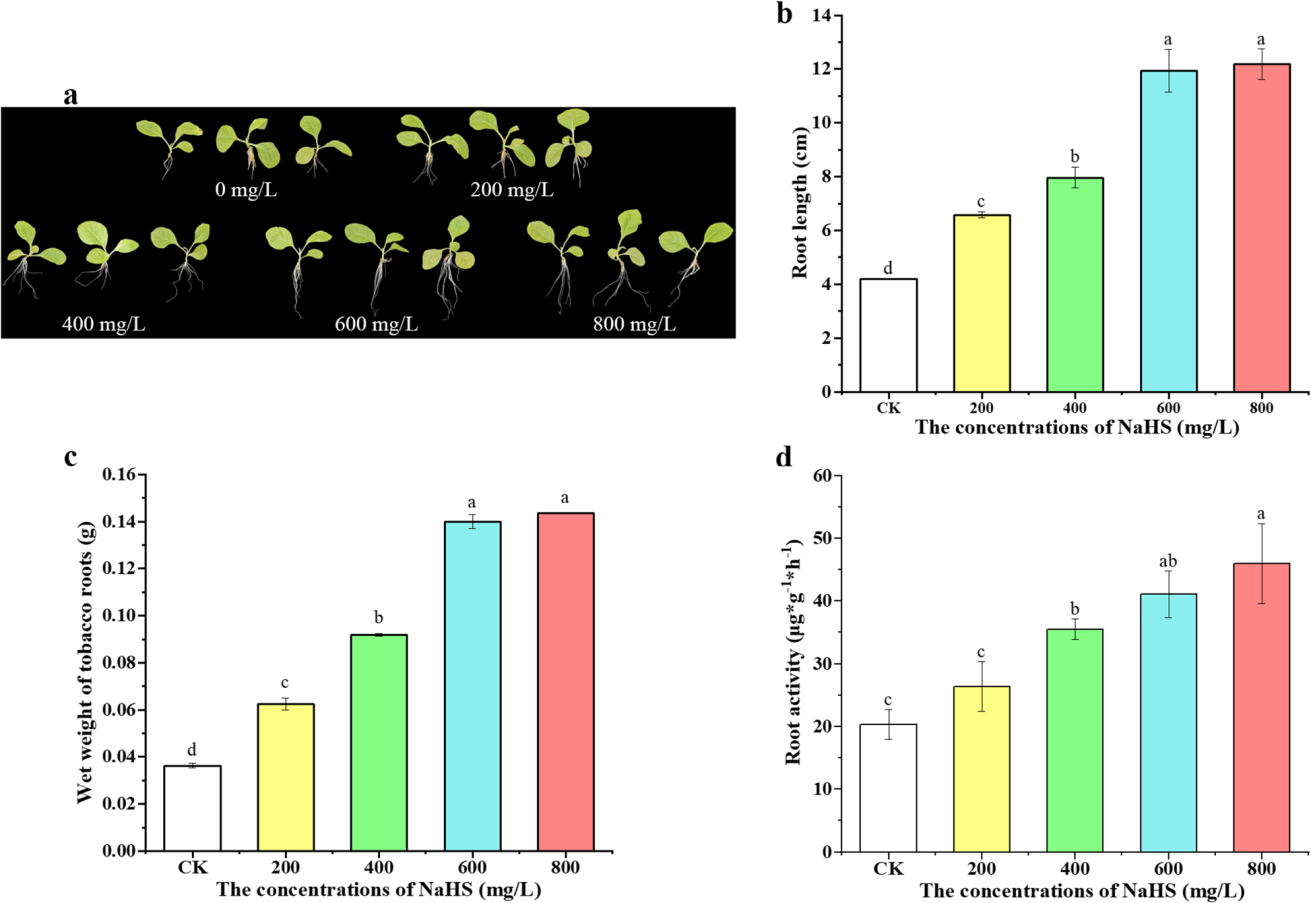
Effects of different NaHS concentrations on tobacco seedlings. **a** Effects of exogenous NaHS effects on tobacco seedling morphology. Tobacco seedlings at the four-leaf stage were treated with water (control) and specific concentrations of NaHS for 10 days. Then the morphology of the tobacco seedling was observed. **b** and **c** Effects of 0, 200, 400, 600 and 800 mg/L NaHS on tobacco root length and root wet weight. **d** Effects of 0, 200, 400, 600 and 800 mg/L NaHS on tobacco root activity after 10 days of treatment. Bars represent mean ± SE. Different lowercase letters indicate statistically significant differences (*P* < 0.05)

To investigate the effect of exogenous NaHS on the growth of tobacco seedlings, seedlings at the four-leaf stage were subjected to different concentrations of NaHS (0, 200 mg/L, 400 mg/L, 600 mg/L, and 800 mg/L) over a 10-day period. While no significant effects were observed on the stem and leaf of the seedlings (Fig. 1a), notable differences were noted in root development. An increase in exogenous NaHS concentration led to a concentration-dependent enhancement in root growth compared to the untreated control (CK, 0 mg/L). The most significant promotion in root length was observed at 600 mg/L NaHS, with higher concentrations (800 mg/L NaHS) showing less effectiveness (Fig. S1).

The effect of NaHS treatment on tobacco root activity was evaluated using the 2,3,5-triphenyl tetrazolium chloride (TTC) staining method after a 10-day period. The root activity significantly increased by 29.5%, 74.4%, 101.8%, and 125.7% with the application of 200 mg/L, 400 mg/L, 600 mg/L, and 800 mg/L of exogenous NaHS, respectively. The higher the concentration, the greater the enhancement in root activity as illustrated in Fig. 1b. However, there was no significant difference in root activity between the 600 mg/L and 800 mg/L treatments, suggesting that 600 mg/L was the optimal treatment concentration to promote root activity. Studies have shown that the exogenous NaHS can improve root activity, but when the concentration is higher, the improvement in activity becomes less obvious.

The study examined the effect of different concentrations of NaHS on the length and wet weight of tobacco roots over a 10-day period. Results indicated a proportional increase in wet weight of tobacco roots with higher NaHS concentrations. Specifically, the maximum wet weight recorded for tobacco roots treated with 800 mg/L NaHS was 0.143 gram (compared to 0.14 gram for 600 mg/L). The longest tobacco root observed under 800 mg/L NaHS treatment measured 12.18 cm (compared to 11.94 cm under 600 mg/L). Additionally, there was a significant increase in root length by 190.10% and wet weight by 296.00% (184.28% and 286.53%, respectively) compared to the control group (CK) (Fig. 1c, d).

### Exogenous NaHS triggers endogenous H_2_S signaling in tobacco roots

**Fig. 2:**
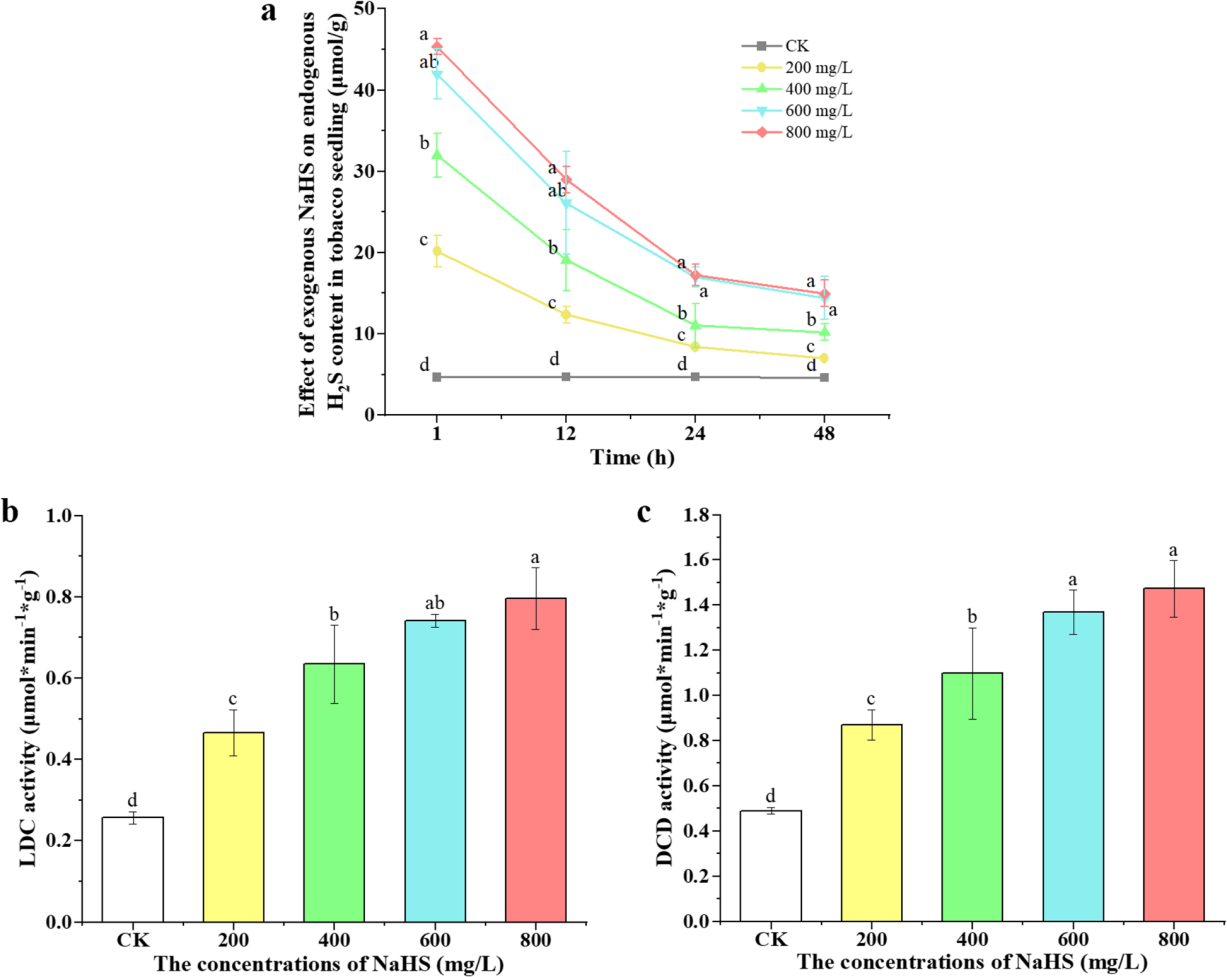
Endogenous H_2_S content of tobacco seedlings treated with 0, 200, 400, 600 and 800 mg/L NaHS for 1, 12, 24 and 48 h respectively (**a**). Effects of 0, 200, 400, 600 and 800 mg/L NaHS on LCD (**b**) and DCD (**c**) enzyme activities. Results are the average of three replicates, and error bars represent standard deviation (SD). Different lowercase letters indicate statistically significant differences (*P* < 0.05)

We investigated the effect of exogenously applied NaHS on the endogenous H_2_S content in tobacco seedlings. The study involved measuring the endogenous H_2_S content and the enzymatic activities of LCD and DCD in tobacco seedlings treated with different concentrations of NaHS (0, 200, 400, 600, and 800 mg/L). NaHS pretreatment significantly increased endogenous H_2_S content compared to the untreated control, with a notable increase of 333.2%, 587.1%, 801.9%, and 874.5% in seedlings treated with 200, 400, 600, and 800 mg/L for one hour, respectively, compared to the control (Fig. 2a). The endogenous H_2_S content in tobacco seedlings stabilized after 24 h. Additionally, no significant difference in endogenous H_2_S content was observed between 24 and 48 h in seedlings treated with 600 and 800 mg/L NaHS. Enzymatic activities of LCD and DCD in tobacco seedlings increased with NaHS treatment, showing a concentration-dependent response (Fig. 2b, c). However, the difference in enzymatic activities between 600 mg/L and 800 mg/L NaHS treatments was not pronounced. The results showed that NaHS could promote the enzymatic activities of LCD and DCD, leading to the degradation of the two cysteine isomers and the production of H_2_S in tobacco under conditions devoid of additional biotic and abiotic stresses.

### Effects of exogenous NaHS on antioxidant enzymes and MDA content of tobacco roots

**Fig. 3:**
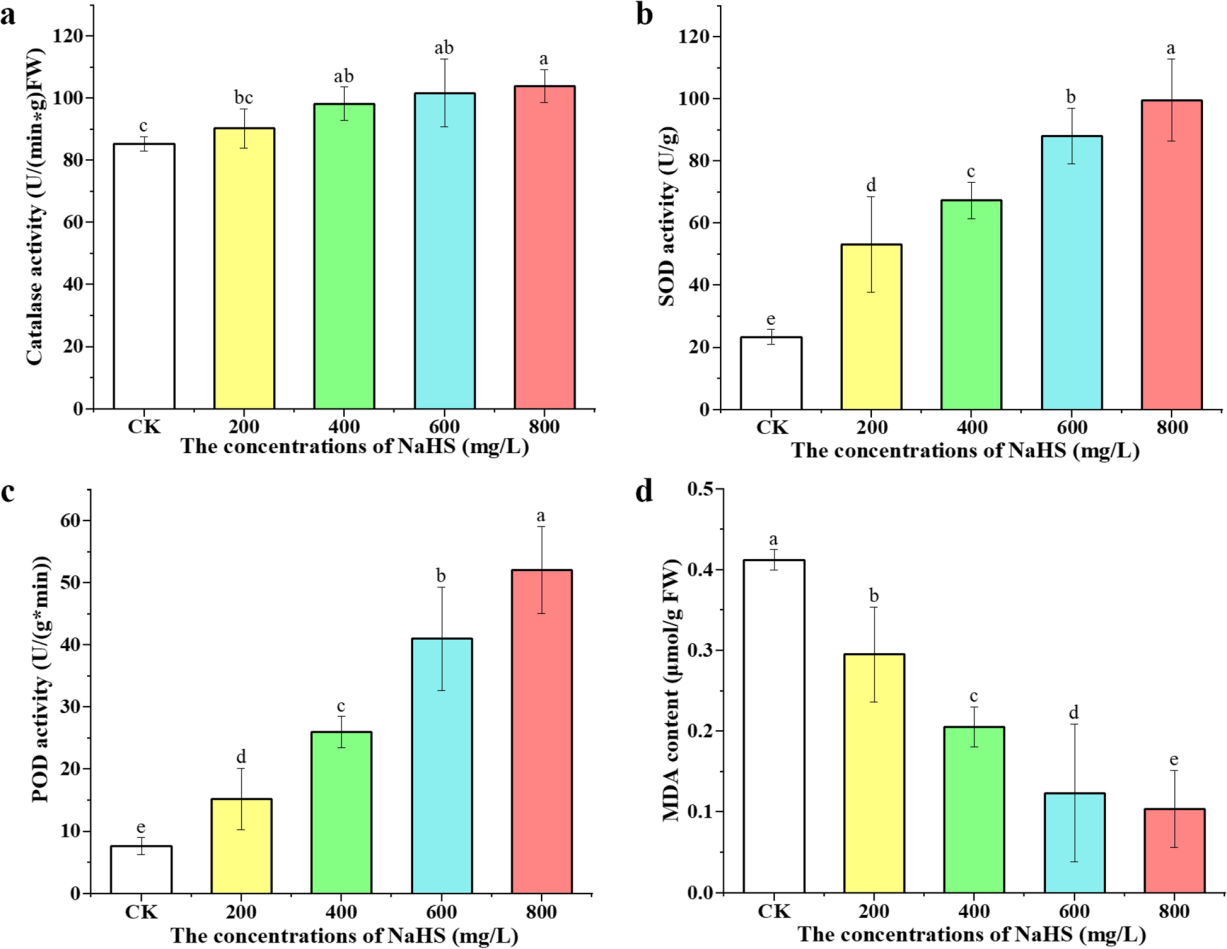
Effects of 0, 200, 400, 600 and 800 mg/L NaHS on CAT activity (**a**), SOD (**b**), and POD enzymatic activity (**c**), and MDA content (**d**) of tobacco seedlings after 10 days of treatment. Bars represent mean ± SE. Different lowercase letters indicate statistically significant differences (*P* < 0.05)

Our study showed that exogenous application of NaHS increased the production of endogenous H_2_S (Fig. 2a), thereby improving plant resistance to various abiotic stresses and ultimately improving plant survival rate [26]. Long-term exposure to abiotic stress can lead to the accumulation of hydrogen peroxide (H_2_O_2_) and ROS, which can damage to lipids, proteins, and cells [15, 31, 32]. Antioxidant enzymes such as CAT, SOD, and POD play an important role in scavenging free oxygen radicals and affecting the stress tolerance of plants [18, 26]. In order to prove the role of NaHS in promoting the CAT, SOD and POD enzyme activities of tobacco roots under non-stress conditions, we irrigated tobacco roots with 200, 400, 600 and 800 mg/L NaHS for 10 days, respectively. Compared to CK, CAT activities increased by 5.8%, 15.2%, 19.2% and 21.8%, respectively. Compared to CK, SOD activities increased by 127.5%, 187.5%, 276.2%, 325.6%, respectively. Compared to CK, POD activities increased by 99.4%, 239.4%, 436.1%, and 580.5%, respectively. Compare to CK, as the concentration of exogenous NaHS increased, the activities of CAT, SOD and POD enzymes gradually increased. It is worth noting that there was no significant difference in CAT activity between tobacco treated with 600 mg/L and 800 mg/L NaHS (Fig. 3a, b, c). In addition, 600 mg/L NaHS treatment significantly increased the activities of CAT, POD and SOD enzymes in tobacco, indicating that this concentration was optimal concentration to improve tobacco stress resistance. Therefore, transcriptomic and metabolomic analyzes were performed in the 600 mg/L NaHS treatment group and CK.

MDA is a byproduct of the peroxidation process of polyunsaturated fatty acids within cells. Elevated levels of free radicals can result in an excessive production of MDA. The concentration of Malondialdehyde is widely recognized as an indicator of oxidative stress and antioxidant levels in plants. In the study, it was observed that the MDA content in the roots of tobacco seedlings decreased significantly compared to CK, as illustrated in Fig. 3d. With increasing NaHS concentrations (200, 400, 600, and 800 mg/L), the MDA content decreased by 28.4%, 50.2%, 70.0%, and 74.9% respectively when compared to CK. These results suggested that the application of exogenous NaHS effectively suppressed the accumulation of MDA in the roots of tobacco seedlings, thereby enhancing the immunity of the seedlings.

### Overview of transcriptome sequencing of tobacco roots after NaHS treatment

**Fig. 4:**
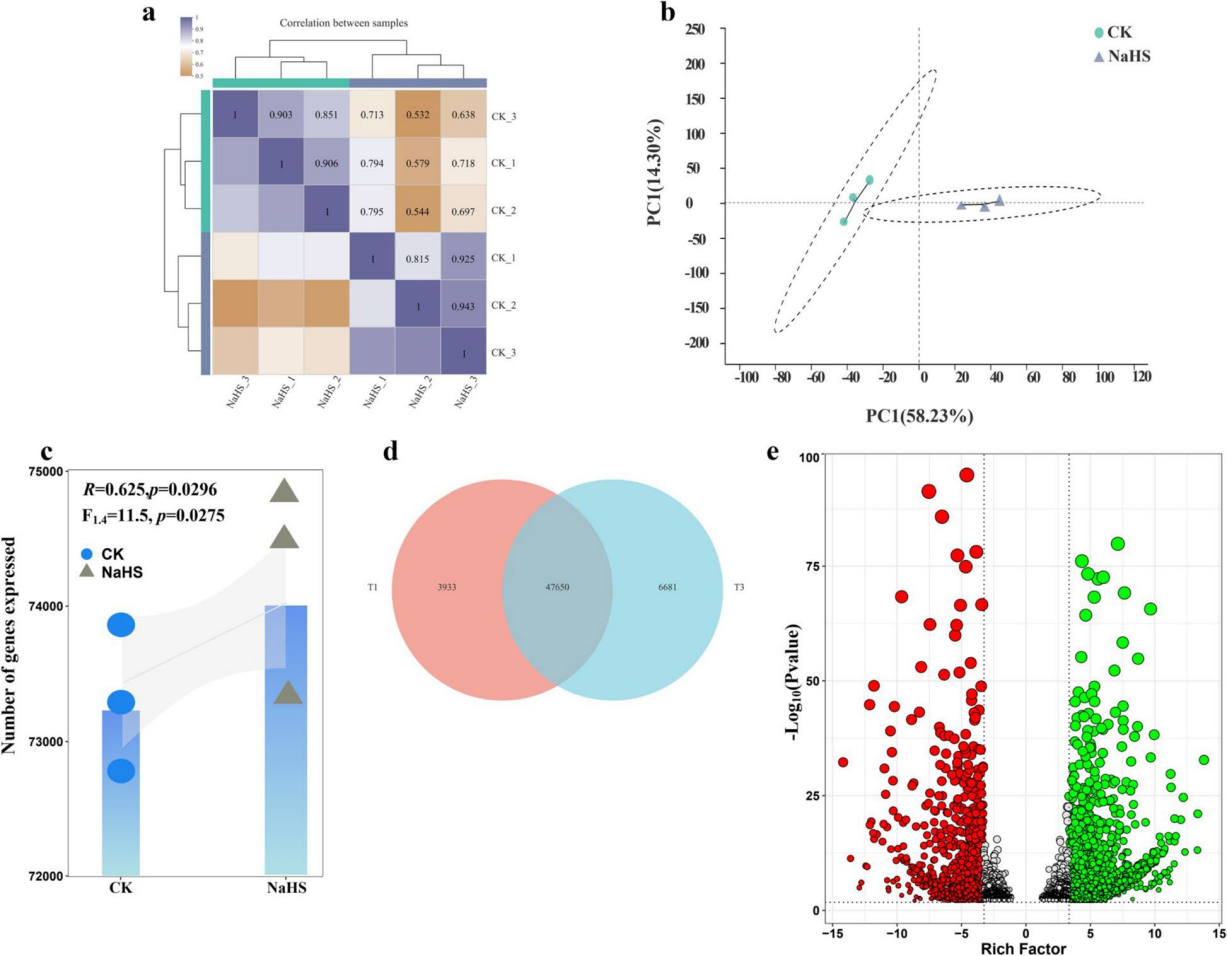
Correlation and PCA analysis of all transcripts. **a** Correlation analysis between samples. **b** PCoA analysis. **c** PERMANOVA analysis. **d** Venn analysis. **e** Volcano plot of the differential expression. The x-axis represents the multiple of the difference, recorded as log2FoldChange. The larger the absolute value, the larger the multiple of the difference. The y-axis represents the significance of the difference, expressed as − log_10_(*P*-value). The larger the value, the more significant the difference

Histological measurements of root systems were conducted to assess gene expression changes in roots exposed to CK and 600 mg/L NaHS treatments. A total of 43.59 Gb nucleotides were generated, including 285,649,662 clean reads, with an average GC content of 43.61%, Q30 > 95.05%, and an underlying error rate of 0.02%, indicating high-quality transcriptome sequencing data (Table S1). Both total reads and uniquely mapped reads exceeded 65% (Table S2), demonstrating the suitability of the chosen reference genome. The correlation heatmap showed that the correlation coefficients of the three treatment groups were above 0.8 (Fig. 4a), and the inter-sample correlation analysis highlighted the high repeatability of the sequencing data from 6 samples. PCA analysis indicated significant variation in unigene expression under 600 mg/L NaHS treatment (Fig. 4b). The combination of PCoA and PERMANOVA based on Bray-Curtis dissimilarity demonstrated that PC1 and PC2 accounted for 72.53% of gene expression frequency, with NaHS exerting a significant effect on the change in gene expression frequency (*R* = 0.625, *P* = 0.0296). Furthermore, NaHS was found to significantly increase gene expression frequency (F1.4 = 11.5, *P* = 0.027) (Fig. 4b, c). After de novo transcriptome assembly, 85,570 expressed unigenes were identified in 6 samples using a threshold of |log2Fold change| ≥ 1 and a strict false discovery rate (FDR) value < 0.05. Venn diagram analysis showed that the number of differentially expressed genes in the CK and NaHS group were 3,933 and 6,681, respectively (Fig. 4d). Subsequently, according to the FPKM (fragments per kilobase per million) value of 3,145 differentially expressed genes in the control/treatment group, 970 genes were down-regulated and 2,175 genes were up-regulated after NaHS treatment (Fig. 4e, Table S3). Table S4 lists the top 10 genes with the highest variance.

### GO enrichment and KEGG pathway analyzes of DEGs

**Fig. 5:**
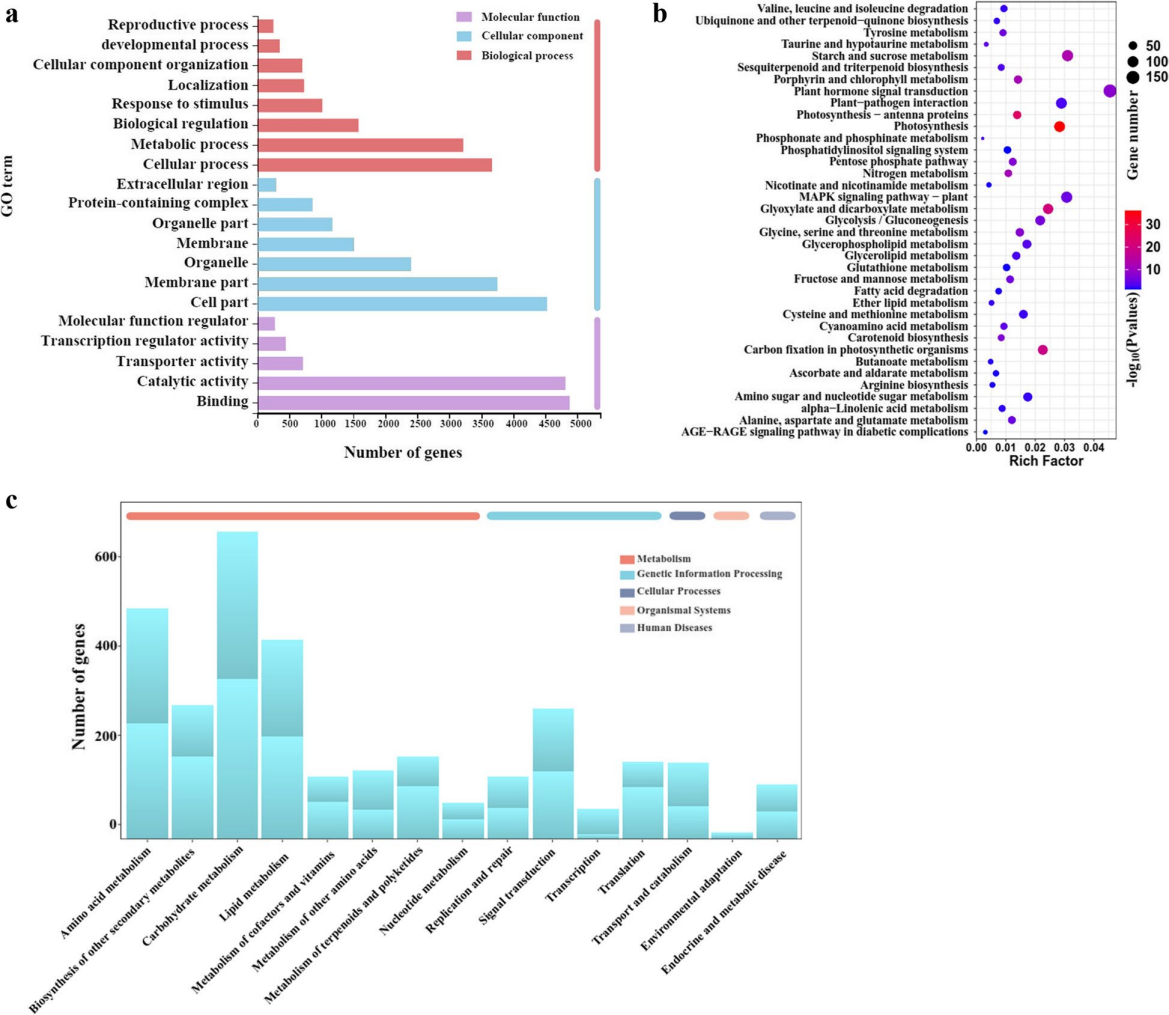
GO and KEGG functional classification of annotated unigenes in tobacco roots. **a** GO annotation analysis of DEGs in CK_vs._NaHS. **b** Enrichment analysis of DEGs in CK_vs._NaHS. **c** KEGG annotation analysis of DEGs in CK_vs._NaHS

Enrichment analyses were conducted in the Gene Ontology (GO) and Kyoto Encyclopedia of Genes and Genomes (KEGG) databases to investigate the biological functions of Differentially Expressed Genes (DEGs) from two comparison groups across three ontologies: biological processes (BPs), molecular functions (MFs), and cellular components (CCs) (Fig. 5a). After NaHS treatment, the root system of tobacco seedlings exhibited enhancements in cell wall biogenesis, oxidoreductase activity, anchored components of the membrane, and supramolecular polymers to bolster plant defense mechanisms (Table S5). A total of 85,570 unigenes were annotated, with 50,547 being up-regulated and 35,023 down-regulated in the CK_vs._NaHS-enriched GO term libraries. The subcategories showing the highest level of enrichment were all related to cellular components. Within the eight components of the functional classification of biological processes, cellular processes and metabolic processes stood out as the most significantly enriched components for differential genes. Notably, differential genes were prominently enriched in cell component categories such as membrane and cellular parts. Regarding the functional classification of molecular function, the enrichment of differential genes associated with binding and catalytic activity was particularly pronounced. Overall, the differential genes were predominantly enriched in components linked to cells, organelles, catalytic activity, and metabolism, indicating a concentration of differentiation genes in secondary metabolic processes.

To classify DEGs based on related signaling pathways and further investigate the differentially expressed genes related to NaHS promoting tobacco root development, we performed KEGG enrichment analysis on these selected differentially expressed genes. The top 20 GO terms with the smallest Q-value were selected for KEGG pathway enrichment analysis (Fig. 5b, Table S6). KEGG annotation analysis identified 128 DEG pathways linked to two treatment stages, with the most common 15 KEGG pathways categorized into metabolism, cellular processes, genetic information processing, environmental information processing, and biosystem. Carbohydrate biosynthesis was the most prevalent, followed by energy metabolism, amino acid metabolism, lipid metabolism, and plant hormone signal transduction. Additionally, KEGG enrichment analysis revealed enrichment in 128 pathways, with 58 pathways showing significant differences. Analysis of KEGG metabolic pathways indicated that DEGs were primarily enriched in 10 major metabolic pathways, with the most notable differences observed in phenylpropanoid biosynthesis, photosynthesis, linolenic acid metabolites, and tyrosine metabolism (Fig. 5c, Table S7). Secondary metabolite biosynthesis pathway was highly enriched, followed by plant-metabolism interaction, plant hormone signaling, plant-plant MAPK signaling, phenylpropanoid biosynthesis, and starch and sucrose metabolism. Consequently, the DEGs related to ‘biosynthetic pathway of secondary metabolites’, ‘plant-metabolism interaction’, and ‘plant hormone signal transduction’ were further investigated.

**Fig. 6:**
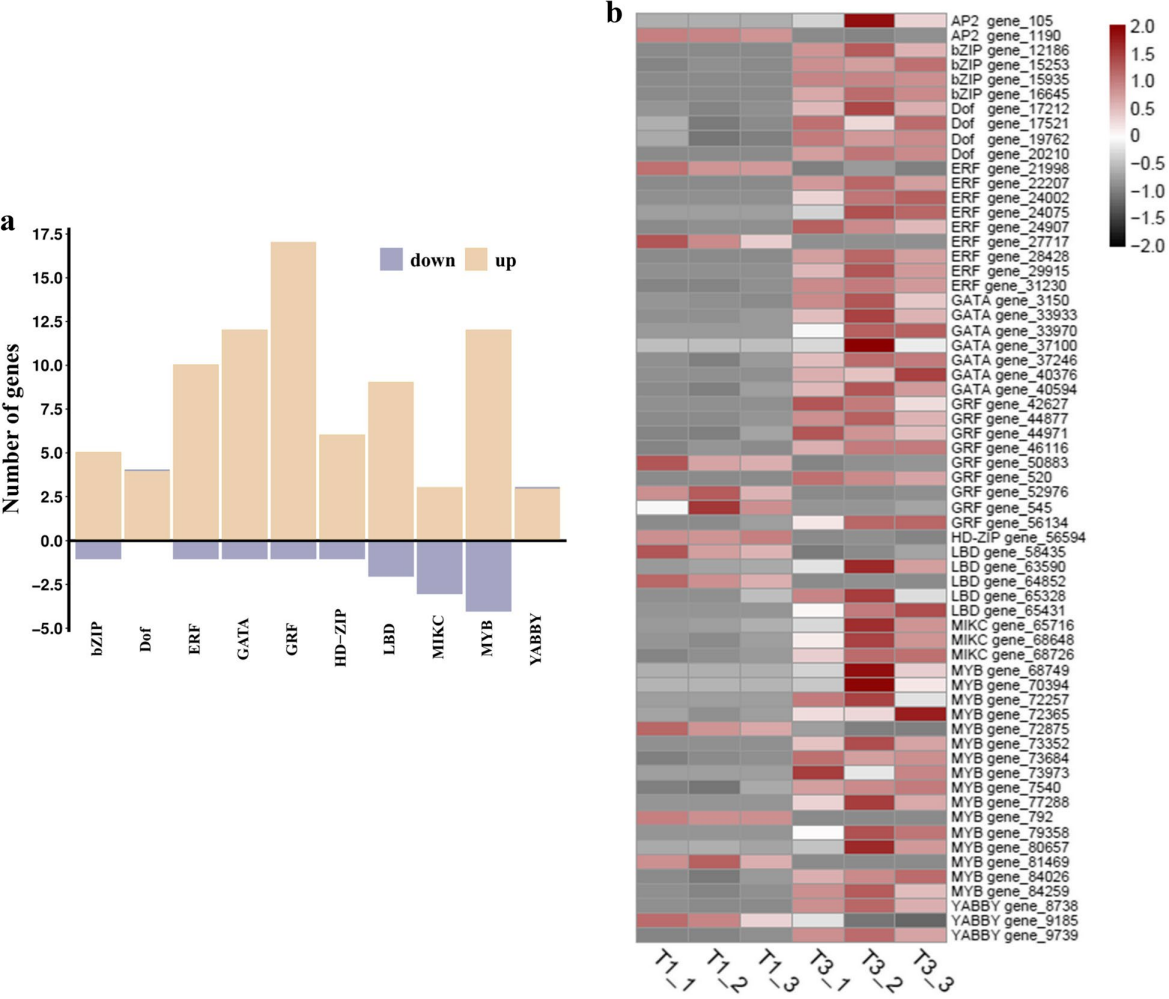
Transcription factor classification and expression profiles. **a** Classification of TFs. **b** Expression profile of relevant TFs. Expressions are normalized. Black represents low expression. Red represents high expression

Transcription factors (TFs) play a crucial role in regulating plant root development. A total of 29 TFs were predicted, with 138 TFs showing significant differences between NaHS_600_ pretreatment and CK treatment in the regulation of tobacco root development. Among these, 112 TFs were up-regulated while 26 were down-regulated. Expression levels of TFs associated with promoting plant growth were analyzed, revealing significant expression of genes related to GRFs (growth-regulating factors), GATAs (TFs recognizing the DNA sequence W-G-A-T-R), MYB TFs (v-myb avian myeloblastosis viral oncogene homologous), and ERFs (ethylene response-factors) in both up-regulated and down-regulated directions (Fig. 6a). In terms of the overall trend of TF gene expression between CK and NaHS, GRF was predominantly up-regulated while MYB was mainly down-regulated (Fig. 6b, Table S8).

### Non-targeted metabolite profiles of tobacco roots after NaHS treatment

**Fig. 7:**
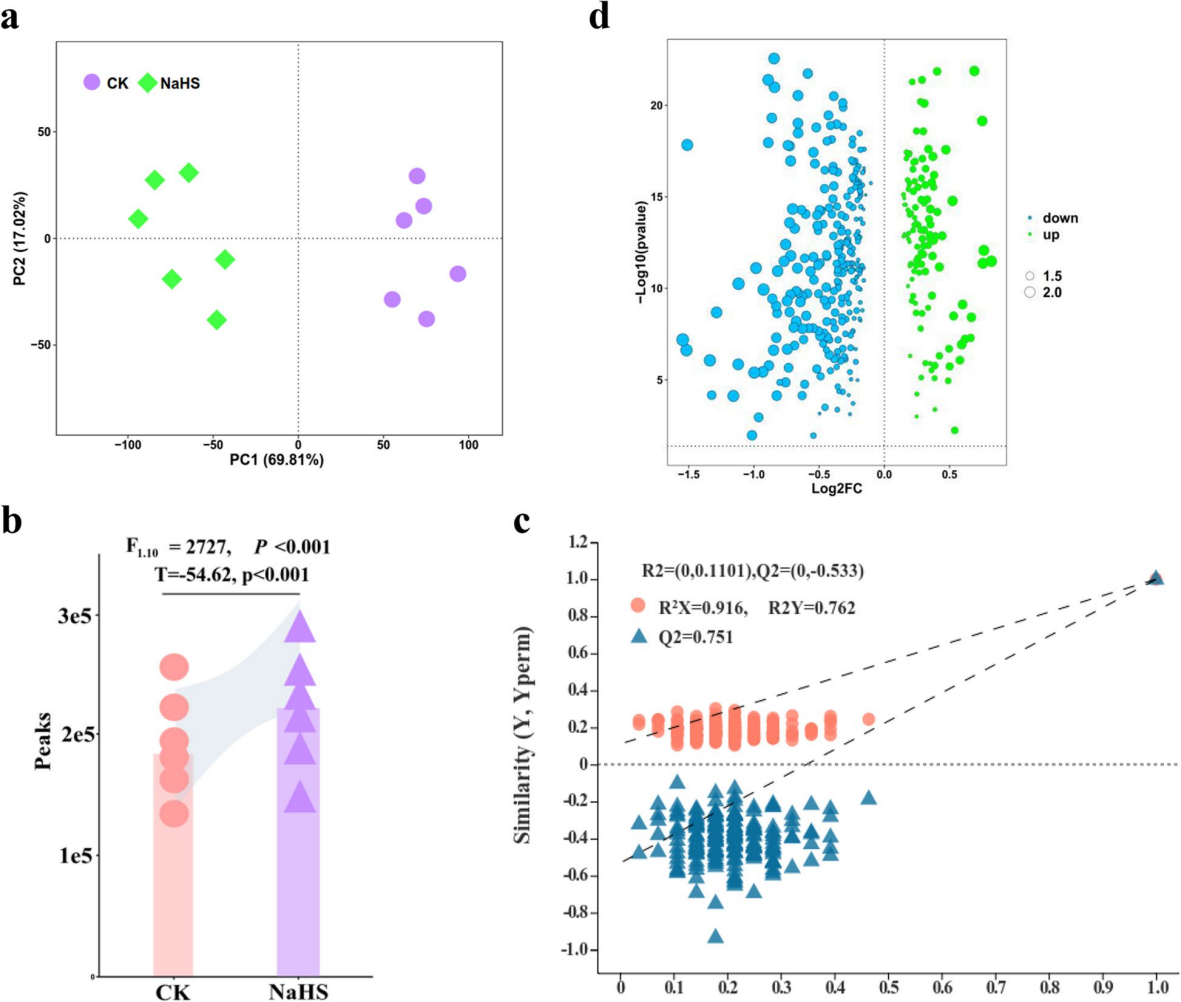
Differences in metabolite accumulation in tobacco roots under NaHS treatment. **a**, **b**, **c** PCA and PLS-DA analysis. **d** Difference statistical volcano plot analysis. The x-axis represents the multiple of the difference, recorded as log2FoldChange. The larger the absolute value, the larger the multiple of the difference. The y-axis represents the significance of the difference, expressed as − log_10_(*P*-value). The larger the value, the more significant the difference

To comprehensively evaluate the effect of NaHS_600_ treatment on tobacco root development, we conducted an untargeted metabolomics analysis on samples treated under identical conditions. A total of 19,891 peaks were detected through UPLC-MS/MS analysis. The peak abundance of NaHS treatment was significantly higher than that of CK, and 821 metabolites were identified using the tandem mass spectrometry (MS2) database. Utilizing PCoA and PERMANOVA based on the microbial dissimilarity index, we found that PC1 and PC2 accounted for 86.83% of the changes in root sediment, underscoring the reliability of the metabolomic data. NaHS treatment significantly affected the composition of root deposits (F1,10 = 2727, *P* < 0.001) (Fig. 7a). Two-way analysis demonstrated the crucial role of NaHS in root sediment composition (F1,10 = 17.991, *P* < 0.001) (Fig. 7b). The permutation test resulted are R^2^ = (0, 0.1101), Q^2^ = (0, -0.533), indicating that the PLS-DA model is not overfitted (Fig. 7c). These indicated that there were significant differences in tobacco roots metabolites between CK and the 600 mg/L NaHS treatment.

To identify the different metabolites resulting from CK and NaHS treatment, we utilized a criteria of Variable Importance in Projection (VIP) value > 1.5 and Fold Change (FC) ≥ 2 or FC < 0.05 to determine the differentially accumulated metabolites (DAMs). In comparison to the CK group, the 600 mg/L NaHS-treated group showed differential enrichment of 384 metabolites, of which 122 metabolites were up-regulated and 262 metabolites were down-regulated (Fig. 7d, Table S9). To further analyze consistent expression patterns of metabolites, we employed the K-means clustering algorithm, which categorized metabolites based on metabolic profile similarities. A total of 10 clusters were identified (Fig. S2), comprising CK_vs._NaHS down-regulated (classes 2, 3, 4, 5, 7, and 8), CK_vs._NaHS up-regulated (classes 1, 6, and 10), and CK_vs._NaHS irregular changes (class 9) categories.

**Fig. 8:**
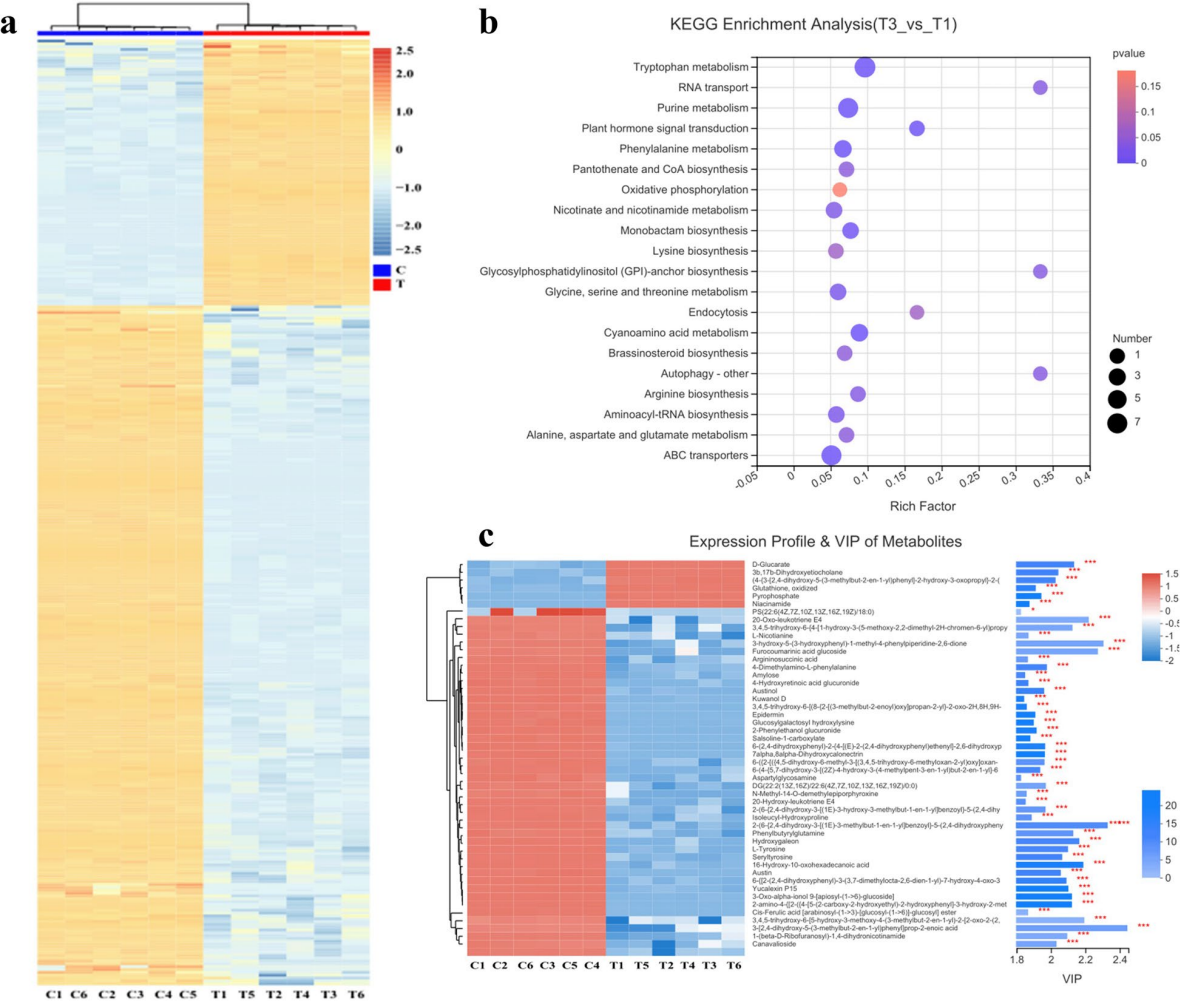
**a** Metabolic heat map. Each column in the figure represents a sample, and each row represents a metabolite. The colors in the graph represent the relative expression of the metabolites in this set of samples. The changing trend of certain expression levels is displayed in the numerical labels below the color bar. Lower right corner. One the left is a dendrogram of metabolite clusters, on the right is the name of the metabolite. The closer two metabolic branches are, the more similar their expression levels are. The top is a dendrogram of sample clustering, and the bottom is the name of the sample. The closer the branches of the two samples are, the more similar the expression trends of the metabolites are. **b** Top 20 KEGG pathway enrichment statistics. The color of the dot indicates the size of the *P*-value. The smaller the q value, the closer the color is to red. The size of the dots represents the number of different genes involved in each signaling pathway. **c** Metabolite cluster tree and metabolite VIP histogram. The metabolite cluster tree is shown on the left. The closer the branches are, the more similar the expression patterns of all metabolites in the sample. Each column represents a sample under the sample name. Each row represents a metabolite, and the color represents the relative expression of the metabolite in the sample group. Shows the correspondence between color gradients and numbers in the gradient block. On the right is a VIP histogram of metabolites, where the column length represents the contribution of the metabolite to the difference between the two groups. The default value is no less than 1. The larger the value, the greater the difference between the two groups. The color of the bar represents the significant difference in metabolites between the two sample groups, that is, the *P*-value. The smaller the *P*-value, the larger the log_10_(*P*-value), and the darker the color. Among them, * represents *P* < 0.05, ** represents *P* < 0.01, and *** represents *P* < 0.001

To identify different metabolites of NaHS during tobacco root development, all metabolites were analyzed using hierarchical cluster analysis method based on Pearson correlation. There was a clear separation between the control and NaHS-treated samples (CK and NaHS). The majority of differential metabolites were identified as fatty acids, carboxylic acids and their derivatives, as well as indoles and their derivatives (Fig. 8a). Subsequent KEGG analysis of the differentially accumulated metabolites highlighted the top 20 enriched pathways, with a focus on GPI anchor biosynthesis (Fig. 8b, Table S10), autophagy, and RNA transport pathways. By applying a threshold of VIP > 1.8 and *P* < 0.05, a total of 50 different metabolites were identified in the CK and NaHS samples (Fig. 8c, Table S11), including compounds such as furocoumarinic acid glucoside, 3-hydroxy-5-(3-hydroxyphenyl)-1-methyl-4-phenylpiperidine-2,6-dione, Austin, and L-tyrosine.

**Fig. 9:**
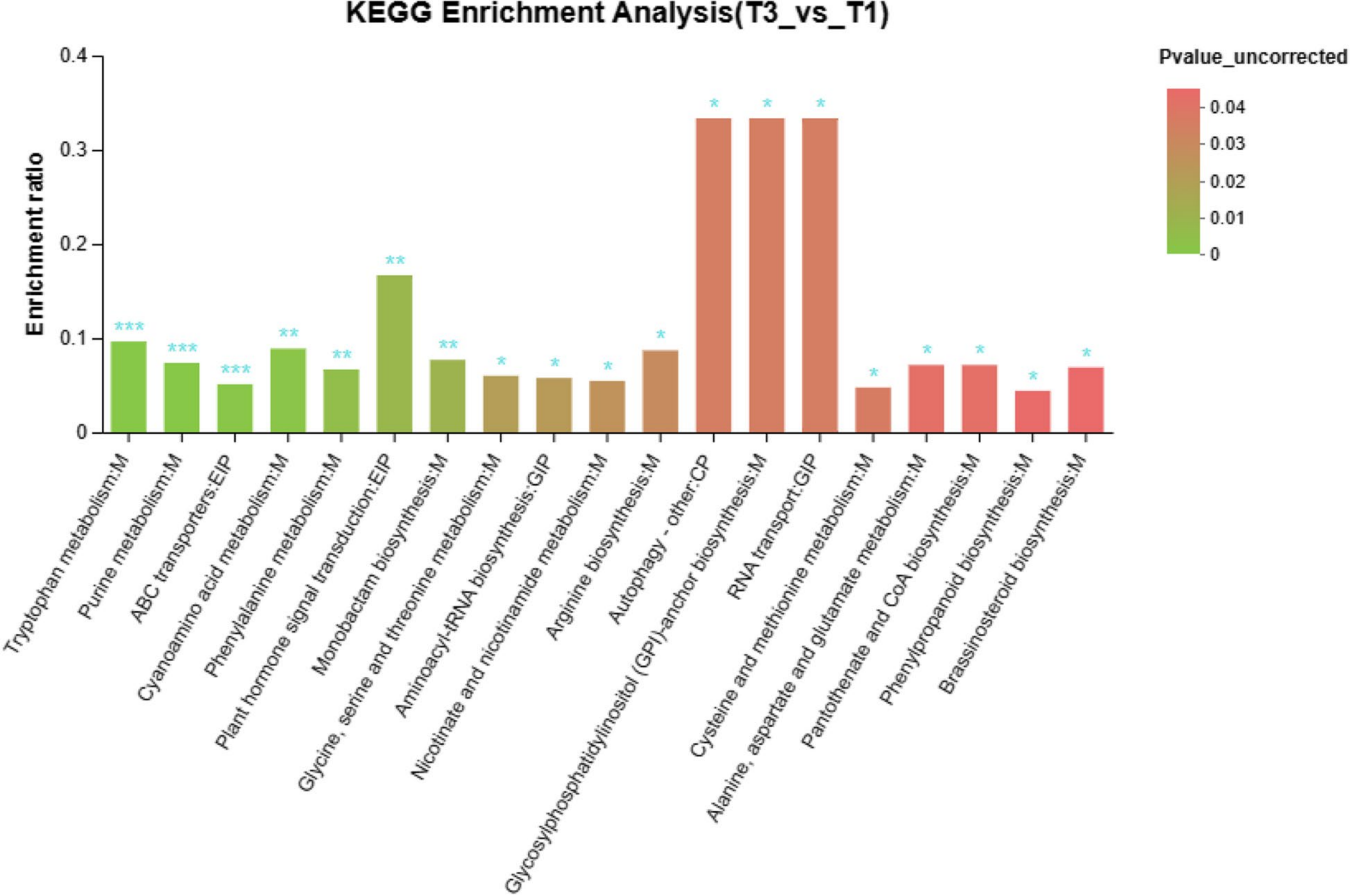
K-means cluster analysis. The abscissa represents the name of the pathway, and the ordinate represents the enrichment rate. The enrichment rate represents the ratio of the number of metabolites enriched in the pathway (number of metabolites) to the number of metabolites related to the metabolic pathway (number of background). The gradient of the column indicates the importance of enrichment. The darker the standard color, the more significant the enrichment of the KEGG term with *P*-value or FDR < 0.001 as ***, *P*-value or FDR < 0.01 as **, *P*-value or FDR < 0.05 is marked with *

**Fig. 10:**
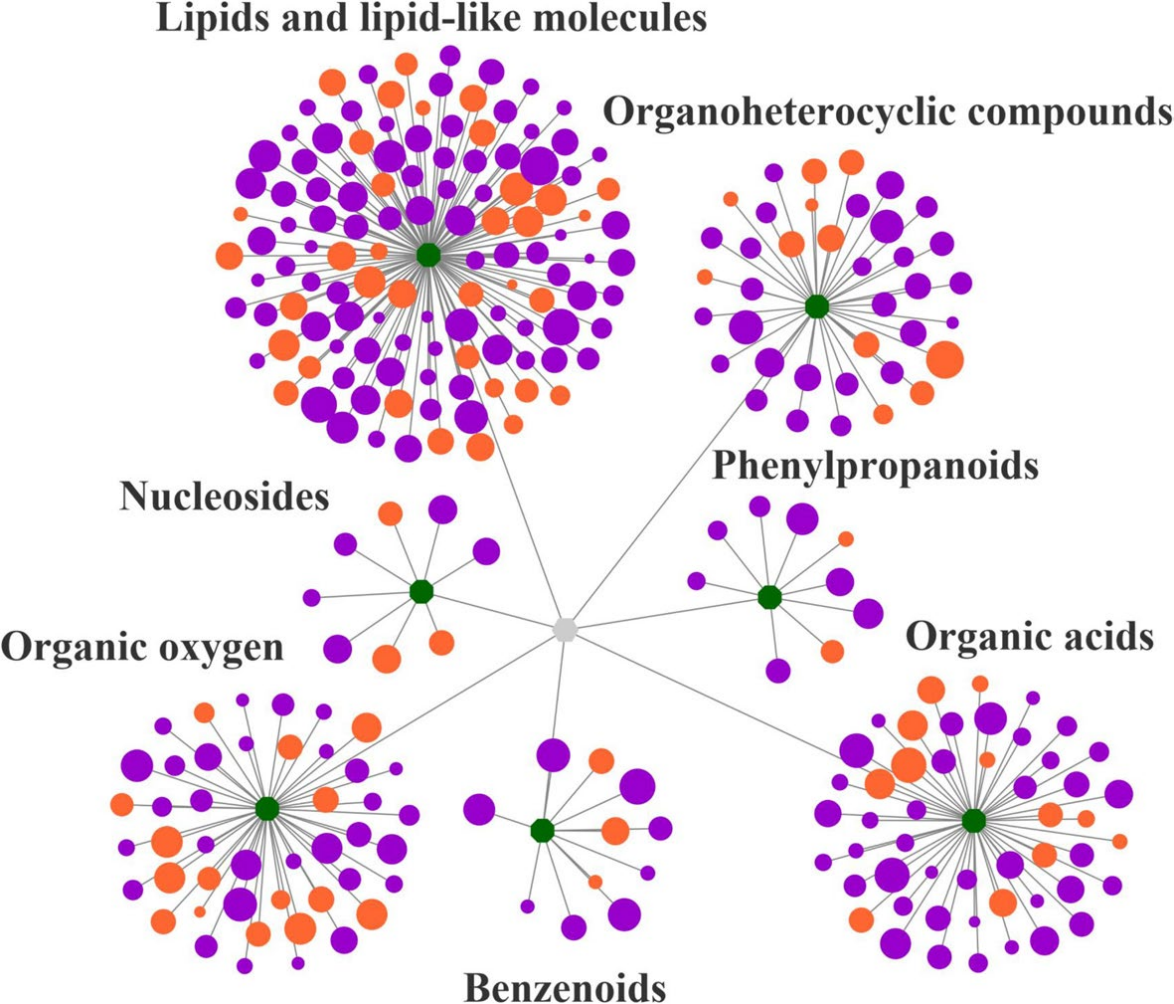
Differential substance cluster network diagram

KEGG analysis of all accumulated metabolites identified 58 enrichment statistics for the KEGG pathway. Significant differences were observed in 19 signaling pathways between the CK and NaHS treatment groups, with tryptophan metabolism, cyanoamino acid metabolism, and plant signal transduction pathways showing the most significance (Fig. 9, Table S10). Lipids and lipid-like molecules were found to be the most abundant categories among all the different metabolites, followed by organic acids and organic oxygen (Fig. 10, Table S12).

### Integrated analyzes of transcriptomic and metabolomic changes involved in vital biological pathways

**Fig. 11:**
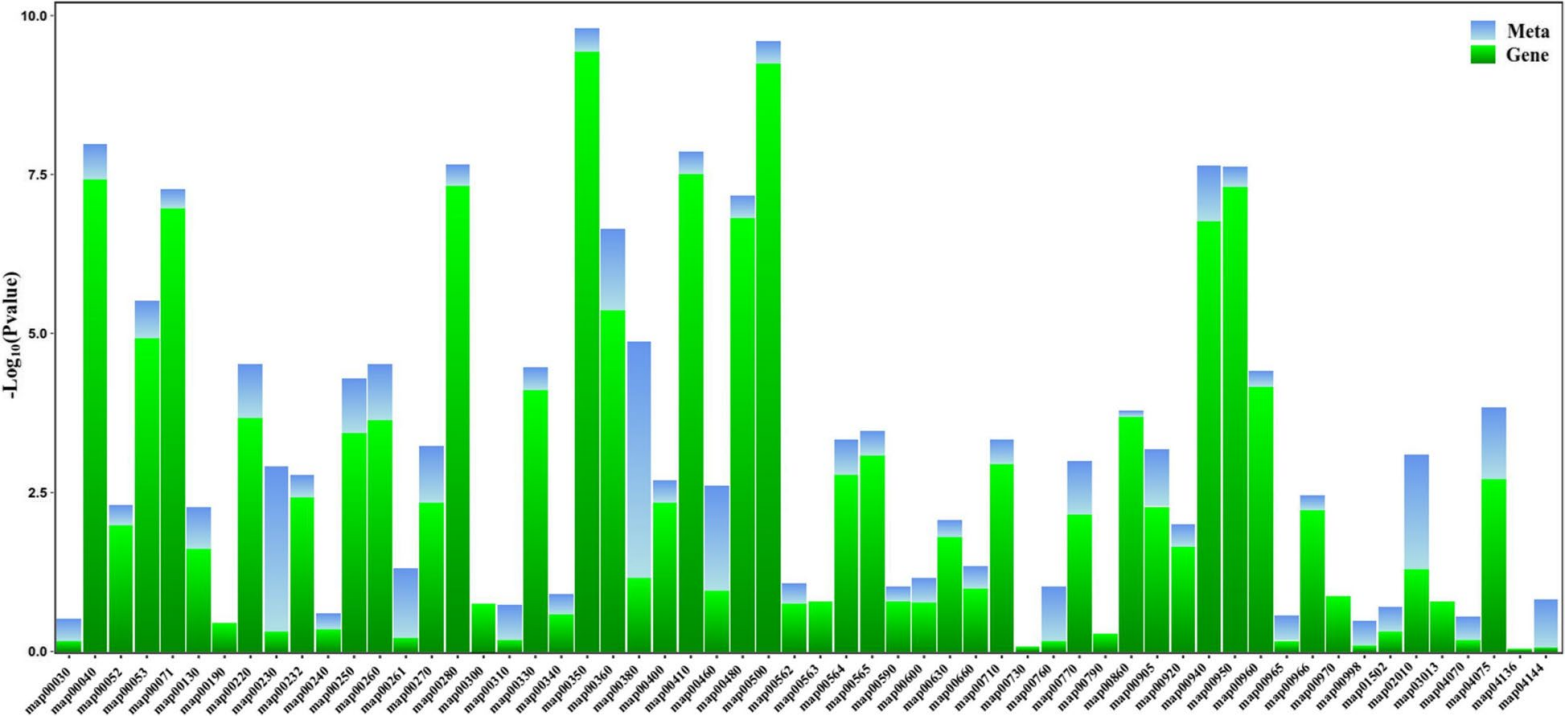
KEGG enrichment analysis after NaHS treatment

The study utilized a combination of metabolomics and transcriptomic data to investigate the effect of CK and NaHS on tobacco roots. By analyzing related genes and metabolites in the treatment groups, correlations were established using the Spearman correlation method. The results revealed that 2175 DEGs showed a positive correlation with 123 DMs (differentially metabolites) (R^2^ < − 0.9 and *P*-value < 0.05), whereas 970 DEGs exhibited a negative correlation with 261 DMs (R^2^ > 0.9 and *P*-value < 0.05). A significant number of cumulative metabolites and DEGs were enriched in 58 KEGG pathways, including pathways such as arginine biosynthesis (map00220), alanine, aspartate, and glutamic acid metabolism (map00250), glycine, serine, and threonine metabolism (map00260), cysteine and methionine metabolism (map00270), phenylalanine metabolism (map00360), and plant hormone signal transduction (map04075). These results suggested that NaHS treatment in tobacco roots could boost root development by leveraging energy metabolism and amino acid biosynthesis mechanisms (Fig. 11, Table S13).

**Fig. 12:**
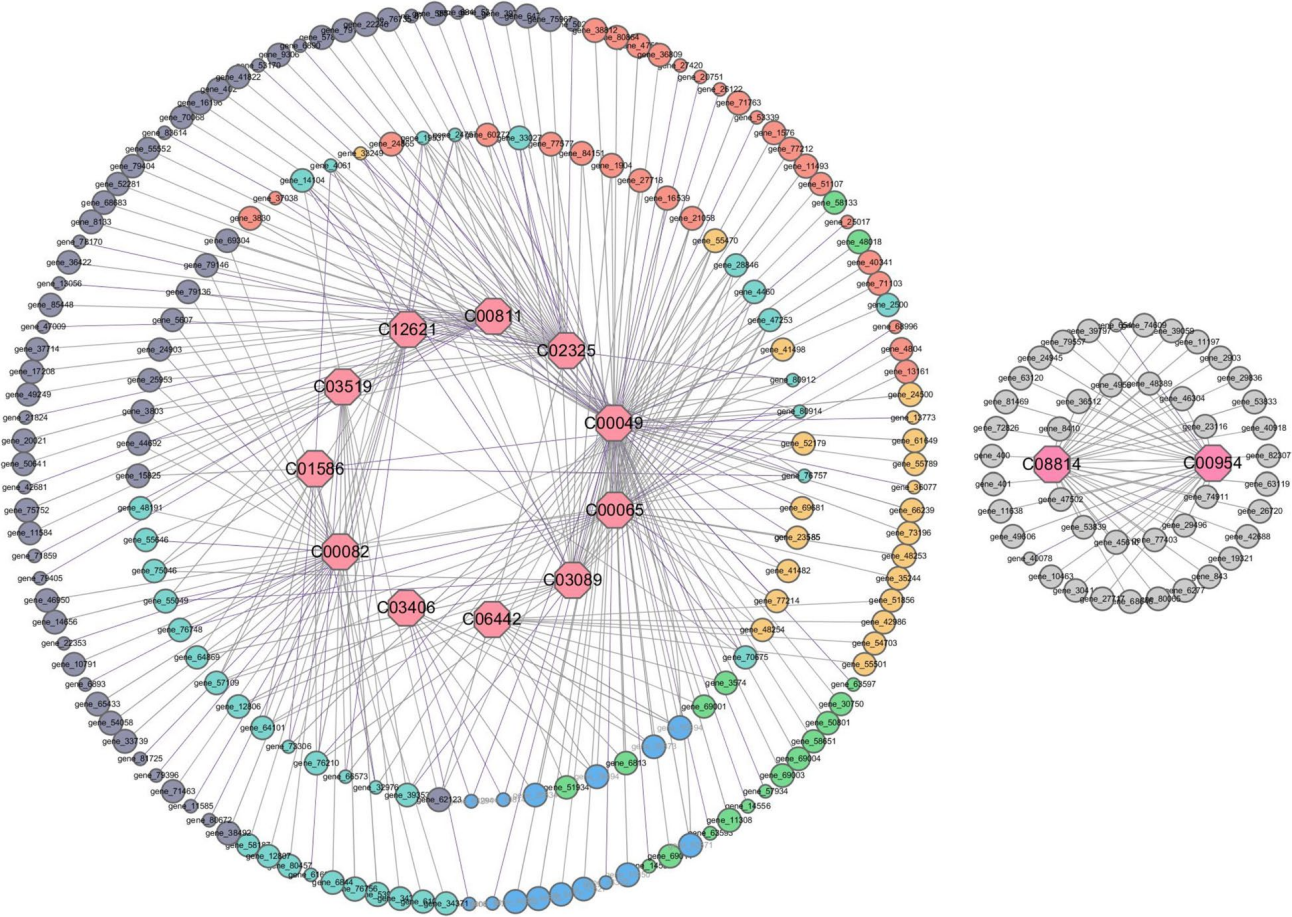
Correlation network analysis of related metabolites and differentially expressed genes in tobacco roots related to NaHS treatment. The gray line represents a positive correlation. The purple line represents a negative correlation

A map of network regulation was constructed by combining different genes in the transcriptome and different metabolites in the metabolome. After NaHS treatment, the differential metabolite aspartate exhibited a positive regulatory relationship with 73 differential genes and a negative regulatory relationship with 30 differential genes. Similarly, the differential metabolite L-serine showed a positive regulatory relationship with 30 differential genes and a negative regulatory relationship with 6 differential genes. Additionally, the differential metabolite brassinosteroid was found to have a positive regulatory relationship with 26 differential genes and a negative regulatory relationship with 2 differential genes. Moreover, the differential metabolite indoleacetic acid demonstrated a positive regulatory relationship with 25 differential genes and a negative regulatory relationship with 2 differential genes. Furthermore, the differential metabolite tyrosine displayed a positive regulatory relationship with 9 differential genes and a negative regulatory relationship with 1 differential gene. Lastly, the differential metabolite 5-methylthioribose was observed to have a positive regulatory relationship with 24 differential genes and a negative regulatory relationship with 4 differential genes (Fig. 12, Table S14).

### Underlying mechanism of the brassinosteroid synthesis pathway that promotes tobacco root development

The study compared and analyzed the enrichment pathways between the transcriptome and metabolome of NaHS-treated tobacco in the KEGG database. Several pathways exhibited differences in enrichment between the CK group and the NaHS-treated group. These pathways include phenylpropanol biosynthesis, brassinosteroid biosynthesis, aspartate and glutamic acid metabolism, plant signal transduction, and arginine and proline metabolism.

**Fig. 13:**
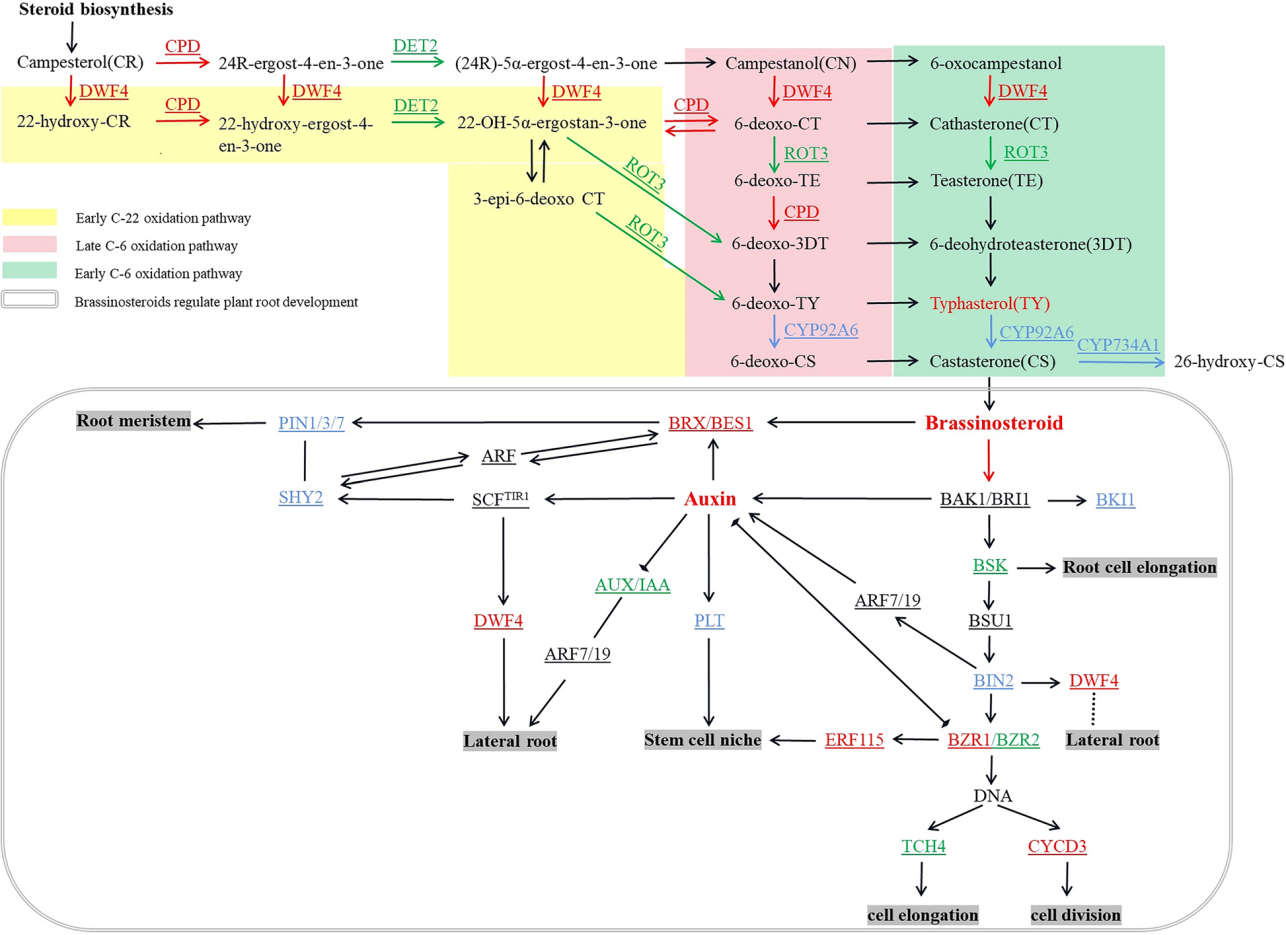
Transcription and metabolic profiles of differentially expressed metabolites and DEGs in the brassinosteroid synthetic pathway under NaHS treatment: Red font: Differentially expressed metabolites or DEGs encoding corresponding enzymes are significantly up-regulated; Green font: Encoding corresponding enzymes, substances or DEGs is significantly down-regulated; Blue font: DEGs encoding the corresponding enzyme are significantly up-/down-regulated; Black font: Genes and enzymes encoding differential metabolites were detected, but no significant differences were found

As the first discovered and most widespread steroidal plant hormone, brassinolide (BL) is a key representative of brassinosteroids (BRs) essential for various stages of plant development. Brassinosteroid signaling begins with the binding of bioactive brassinosteroid to receptors on the cell membrane, initiating a cascade of effects that regulate the expression of genes related to plant growth and morphogenesis. The biosynthesis of brassinosteroid involves three pathways: early C-6 oxidation, late C-6 oxidation, and early C-22 oxidation pathways [33–36]. In order to further elucidate the specific role and regulatory mechanism of NaHS, we conducted a comparison and analysis of KEGG enrichment pathways in the transcriptome and metabolome of tobacco roots treated with CK_vs._NaHS_600_. After NaHS pretreatment, various metabolic pathways were enriched, including phenylpropanol biosynthesis, BL biosynthesis, aspartate and glutamic acid metabolism, plant signal transduction, and arginine and proline metabolism. The results revealed that two significantly different metabolites encoded by 26 significant DEGs were associated with brassinosteroid biosynthesis (C08814; c15793) and BR signaling (Fig. 13).

BL is a C28 BR whose synthesis begins with conversion of campestanol (CN) via the early or late C-6 oxidation pathway, depending on whether the BR contains a ketone group at the C-6 position (the former) or a deoxy form (he latter) (Fig. 13). These parallel pathways converge on castasterone (CS), the direct precursor of BL, catalyzed by the C-22 hydroxylase DWF4 (dwarf 4). Furthermore, CPD (cytochrome P450 superfamily protein), DWF4, DET2 (3-oxo-5-steroid 4-dehydrogenase family protein), and ROT3 (3-epi-6-deoxocathasterone 23-monooxygenase) can catalyze the multistep C-22 hydroxylation reaction and participate in an alternate BR synthesis pathway known as the early C-22 oxidation pathway [37, 38]. An analysis of KEGG enrichment pathways comparing the transcriptome and metabolome of tobacco roots treated with CK vs. NaHS revealed that genes encoding CPD (cytochrome P450 superfamily protein) and DWF4 were up-regulated, while genes encoding DET2 (3-oxo-5-steroid 4-dehydrogenase family encoding protein) and ROT3 (3-epi-6-deoxocathasterone-23-monooxygenase) were down-regulated after NaHS pretreatment. As a result, the levels of end metabolites such as typhasterol (TY) and BRs were increased.

Upon transmission of the BR signal, BL is up-regulated. This up-regulation leads to the binding of BL to the extracellular region of the BRI1 protein, initiating phosphorylation of the intracellular kinase region of BRI1. Subsequently, the activated BRI1 phosphorylates the membrane binding protein BSK (BR signaling kinase), resulting in the down-regulation of the BSK gene family. The activator protein phosphatase PP1-type phosphatase BSU1 (BRI1 suppressor 1) is then activated by BSU1 [39], which acts on BIN2 to down-regulate the BIN2 gene. The down-regulated TFs BZR1 and BZR2/BES1 (brassinazole-resistant 2/BRI1-EMS-suppressor 1) are then rapidly dephosphorylated by PP2A (protein phosphatase 2 A) [36], allowing them to enter the nucleus, bind to target genes, and regulate transcription.

Xyloglucan endotransglucosylase/hydrolase (XTH) is a group of enzymes, comprising xyloglucan endotransglucosylase (XET) and xyloglucan endohydrolase (XEH), that play crucial roles in modifying cell walls. TOUCH4 (TCH4), also known as XTH22, encodes a protein with XET enzyme capabilities. The expression of TCH4 is rapidly up-regulated under the effect of brassinosteroids, auxin, and various environmental stimuli such as touch, temperature shock, and darkness [40]. After NaHS treatment, the TCH4 gene was down-regulated, leading to cell elongation and greatly promoting root development. Plant development is accompanied by cell division, and cell cycle activity can regulate cell division [33]. In plants, the expression level and activity of CYCD (D-type cyclin) are affected by hormones and carbohydrate concentrations, which have an important impact on the cell division process of plants [41]. Most studies on CYCD have focused on the functions of individual CYCD proteins, mainly based on their effects on plant growth and development. CYCD3 gene expression is up-regulated, cell division is accelerated, and root elongation is accelerated.

Previous studies have shown that the BR signaling pathway frequently interacts with auxin to regulate the growth and development of root meristems. The stem cell niche consists of QC cells (quiescent center) and surrounding stem cells. QC cells are important for maintaining the niche stability and growth status of stem cells. BZR1-mediated BR signaling activates ERF115, and the ERF115 gene is up-regulated, thereby up-regulating the expression of PSK5 and promoting QC cell division [42]. Auxin can induce the expression of PLETHORA (PLT) TFs and regulate QC cell division, thereby regulating root growth.

The root meristem is a critical region for root development, continuously reproducing and differentiating. The regulation of BR exhibits characteristics of spatial-temporal balance and hormone interaction balance [34]. Research indicated that the BR signal sensed by BRI1 in the root epidermis could stimulate auxin expression in the root meristem, leading to its proliferation, while the BR signal in the stele activates the activities of BAK1 (BRI1-associated receptor kinase 1), BRL1, and BRL3, promoting differentiation [43]. SHY2 (short hypocotyl 2) acts as a repressor of auxin signaling, while BRX (brevis radix) is involved in the BR signaling pathway. Both SHY2 and BRX play crucial roles in the crosstalk between BR, auxin, and cytokinin (CTK) in regulating root meristem growth and development [44, 45]. The early development of the root meristem is affected by hormonal balance, with BR and auxin being up-regulated (Fig. 13). TFs control root meristem development by activating SHY2 expression and inhibiting PIN expression. BRX can be induced by large amounts of IAA, while BR has a slight inhibitory effect on it. By competing with SHY2, BRX becomes dominant and can temporarily increase PIN3 expression. Both regulate root meristem development [46, 47]. As shown in the figure, the BRX gene is up-regulated, and the SHY2 family is both up-regulated and down-regulated. BRX dominates, enhances PIN3 and promotes root meristem development.

**Fig. 14:**
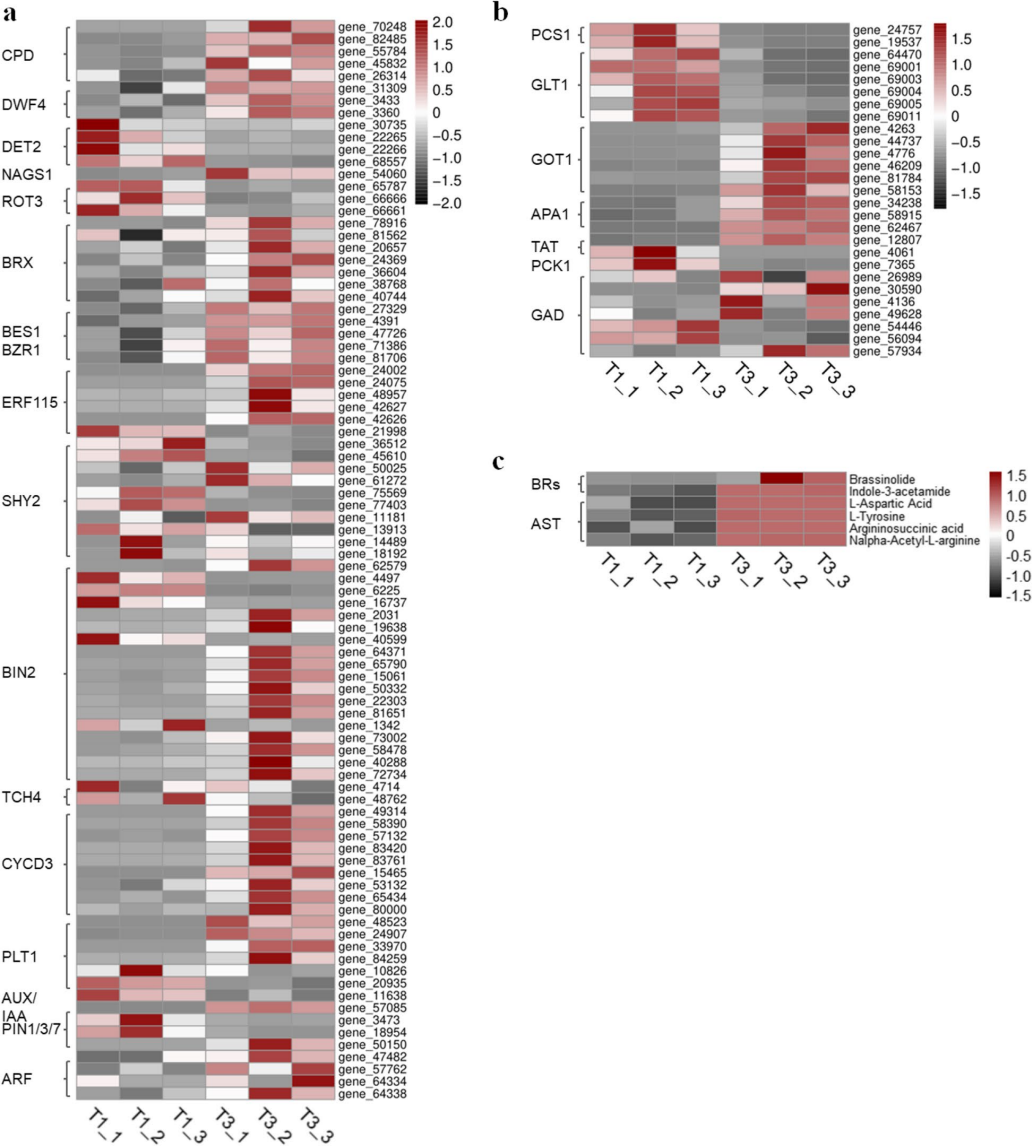
Heat map of DEGs-enriched brassinosteroid biosynthesis pathway (**a**), aspartate metabolism pathway (**b**), and various metabolites in the brassinosteroid synthetic pathway and aspartate metabolism pathway under NaHS treatment (**c**)

Plant lateral roots play an important role in absorbing water and nutrients from the environment, contributing significantly to plant growth. The growth and development of lateral roots are jointly regulated by auxin and BR (Figs. 13 and 14a). Early studies have shown that low concentrations of BR promote lateral root development by increasing polar auxin transport. On contrast, high concentrations of BR inhibit the formation of lateral roots [35, 48]. DWF4 (dwarf 4) is an important regulator of the BR synthesis pathway. In addition, BIN2 (BR-insensitive 2) is a negative regulator of BR signal and has a positive impact on lateral root development. After NaHS treatment (Table S15), the BIN2 gene was down-regulated, suggesting that BIN2 may be involved in the regulation of DWF4, and the specific mechanism needs further study. Up-regulated BR facilitates the degradation of AUX/IAA (auxin/indole-3-acetic acid), such as IAA14/SLR (indole-3-acetic acid 14/solitary root) single root, essential for lateral root development by modulating the transcription levels of the auxin input carriers AUX1 (auxin 1), LAX2 (like-aux1 2), and LAX3 (like-aux1 3) in roots. The depletion of AUX/IAA further activates ARF7/ARF19 (auxin response factor 7/auxin response factor 19), thereby promoting lateral root development.

### The aspartate metabolic pathway may also play an important role in tobacco root development

Aspartate serves as the initial compound in the aspartate metabolic pathway, originating from the transamination of phthalic acid. It acts as a key precursor in two primary pathways. In one pathway, it leads to the formation of aspartate, crucial for nitrogen transport and storage in plants. The synthesis of aspartate involves the transfer of amino compound nitrogen from glutamate to aspartate salt, a process catalyzed by aspartate synthase. This pathway also serves as an alternative route for the synthesis of lysine, threonine, methionine, and isoleucine.

**Fig. 15:**
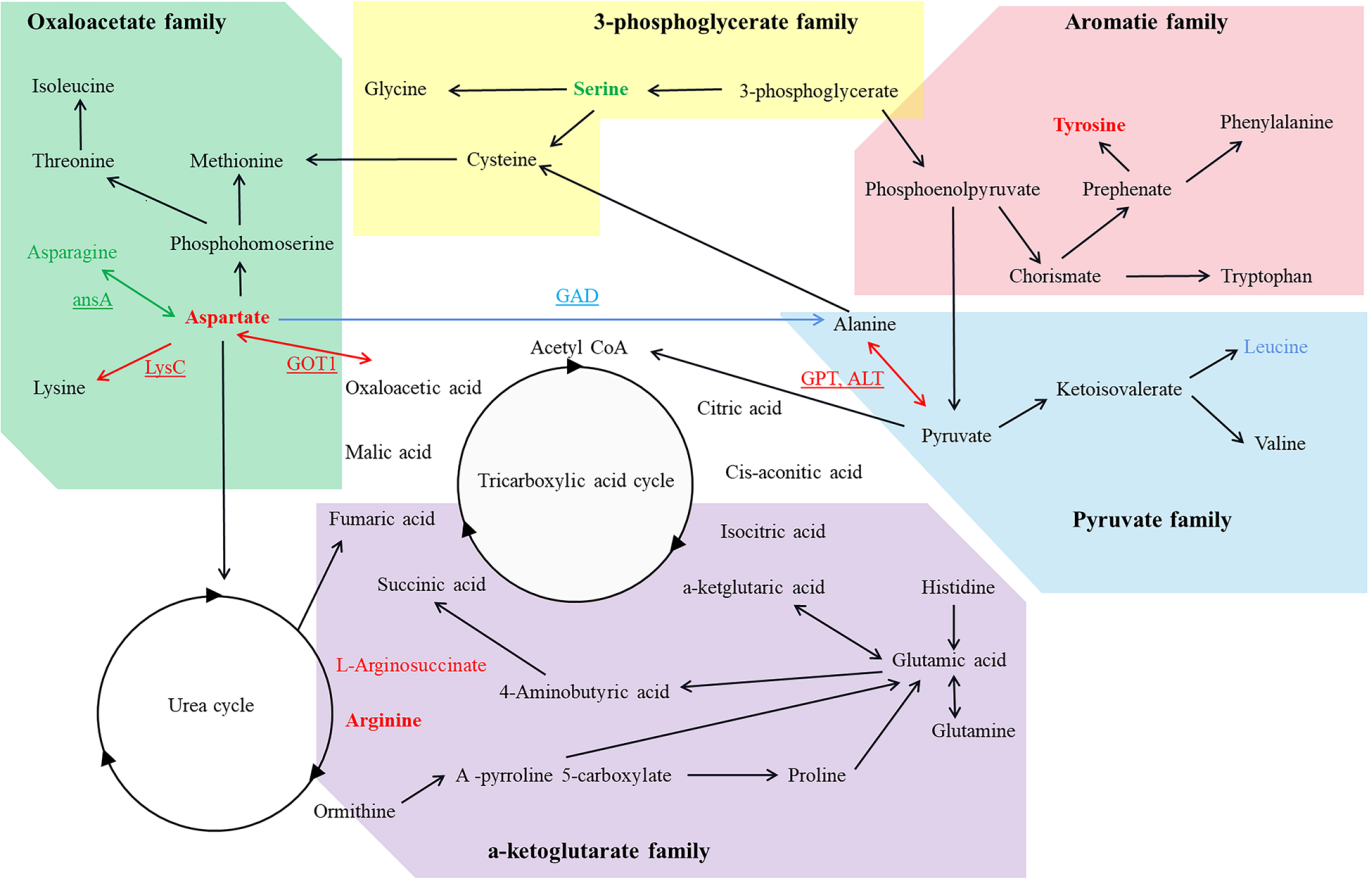
Transcriptional and metabolic profiles of significantly different metabolites and DEGs in the aspartate pathway under NaHS treatment

After treatment with NaHS, plants undergo modifications in certain amino acids by altering amino acid metabolic pathways, which helps in maintaining the stability of functional proteins and metabolic enzymes. Plant proteins and enzymes are composed of amino acids falling into five categories: 3-phosphoglycerate, α-ketoglutarate, pyruvate, oxaloacetate, and aromatic amino acids derived from the carbon framework [49]. The collective analysis results indicate that the use of hydrogen sulfide leads to significant changes in the amino acid content of tobacco roots (Fig. 15). Different treatments efficiently distinguished the content of various amino acids in tobacco roots: the levels of α-ketoglutarate-arginine were notably increased, while asparagine of the α-ketoglutarate group, serine of the 3-phosphoglycerate group, and aromatic tyrosine were significantly decreased. Arginine, an amino acid with a high nitrogen to carbon ratio, serves not only as a protein component but also as a precursor for the biosynthesis of nitric oxide, polyamines, and proline [50]. The substantial accumulation of arginine in tobacco roots can enhance the development of tobacco roots, thereby effectively promoting the growth and development of tobacco.

**Fig. 16:**
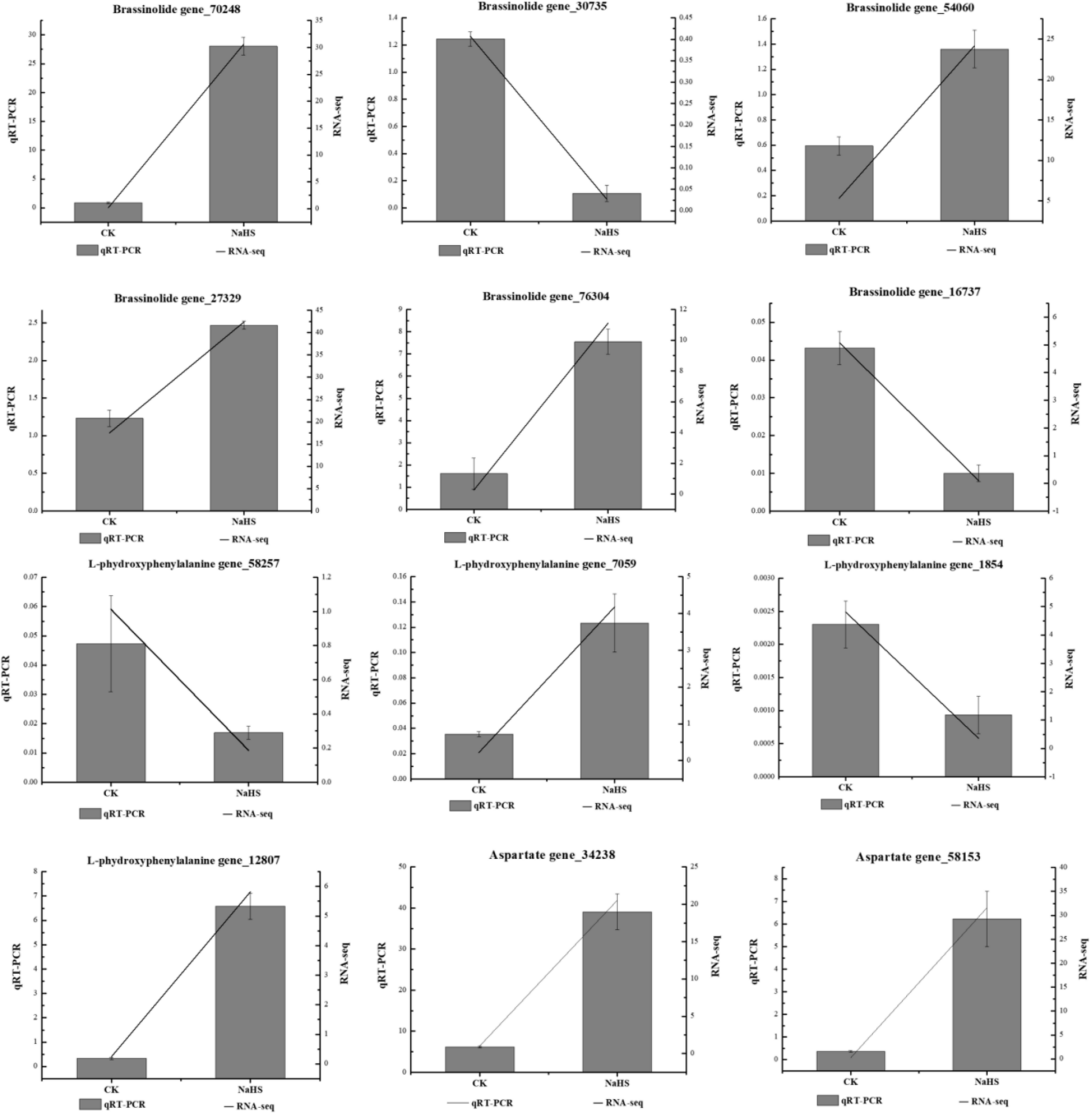
Quantitative real-time fluorescence PCR results

The intricate physiological and biochemical processes of amino acids in plants involve transamination, deamination, and various reaction pathways leading to the production of carbohydrates and organic acids through metabolic pathways like the citric acid cycle and glycolysis. Ultimately, these compounds are converted into proteins or other cellular components within plants. The complex interplay between amino acid content and gene expression warrants further investigation. Analysis of genes associated with amino acid synthesis revealed significant changes in the expression of DEGs related to aspartate (Fig. 14b, Table S16), such as *GOT1* (glutamate oxaloacetate transaminase 1) and *ansA* (aspartate synthesis gene, L-asparaginase), highlighting the crucial role of aspartate. Aspartate serves as a key precursor in two main pathways, effecting glutamine production and providing amino donors for the synthesis of other amino acids, purines, and pyrimidines. This amino acid plays a vital role in growth and development. Validation of transcriptome data through quantitative real-time PCR (qRT-PCR) experiments on genes involved in amino acid and organic acid synthesis and metabolism demonstrated consistency between the two datasets (Fig. 16).

### Preliminary review of some key genes involved in the brassinosteroid synthetic pathway and the aspartate metabolic pathway

To validate the accuracy of the RNA sequence data, qRT-PCR was employed to examine the associated genes of 12 differential metabolites, including brassinosteroid, aspartate, and phydroxyphenylalanine (Fig. 16). The qRT-PCR results verified that the expression patterns of the 12 DEGs closely matched the transcriptome data, thus reinforcing the reliability of the RNA sequence data.

The key genes involved in brassinosteroid metabolites *CPD* (cytochrome P450 superfamily protein, Gen_70248), *NAGS1* (N-acetyl-L-glutamate synthase 1, Gen_54060), *BZR1* (brassinazole-resistant 1, Gen_27329), and *BRI1* (brassinosteroid-insensitive 1, Gen_76304, leucine-rich receptor-like protein kinase family) exhibited an up-regulation trend, with NaHS treatment showing significantly higher expression levels compared to CK. Conversely, *DET2* (3-oxo-5-steroid 4-dehydrogenase family protein, Gen_30735) and *BIN2* (BR-insensitive 2, Gen_16737) displayed a down-regulation trend, with NaHS treatment showing significantly lower expression levels than CK. Additionally, the key genes *APA1* (aspartic protease, Gen_34238) and *PCS1* (phytochelatin synthase 1, Gen_58153, eukaryotic Aspartyl Protease family protein) exhibited an upward trend post-treatment, with NaHS treatment resulting in significantly higher expression levels compared to CK. Furthermore, tyrosine metabolite key genes *ACR12* (ACT domain-containing protein, Gen_58257) and *PPC3* (phosphoenolpyruvate carboxylase 3, Gen_1854) displayed a downward trend, with NaHS treatment resulting in significantly lower expression levels compared to CK. Finally, *AT1G* (phosphoenolpyruvate carboxylase family protein, Gen_7059) and *TAT* (tyrosine transaminase family protein, Gen_12807) exhibited an upward trend post-treatment, with NaHS treatment showing significantly higher expression levels than CK.

## Discussion

Numerous studies have shown that H_2_S functions as a signaling molecule in various physiological processes in plants. Optimal levels of H_2_S are essential for processes such as root formation and growth, overall plant development, and responses to both biotic and abiotic stresses [6, 11, 15, 17, 18, 31, 32, 51]. Several reports have highlighted that H_2_S can inhibit ROS production and enhance antioxidant activity of plants under environmental stresses such as heavy metals, cold, drought, and salinity [7, 9, 15, 18, 31, 32]. For example, NaHS has a dose-dependent effect on the formation and growth of sweet potato (*Ipomoea batatas* L.) lateral roots [11]. Li demonstrated that pretreatment with NaHS increased endogenous H_2_S levels in *Malus hupehensis* seedling roots, resulting in increased root activity, length, and antioxidant enzyme activities (CAT and POD), while reducing MDA content and detoxifying ROS induced by alkaline salt stress [52]. Similarly, NaHS pretreatment increased antioxidant enzyme activities (CAT, POD, and SOD), leading to decreased MDA and H_2_O_2_ levels in cucumber roots under salt stress [53]. H_2_S was found to sustain and enhance SOD and POD activities in tomato plants [31]. The current study revealed that tobacco seedlings treated with exogenous NaHS exhibited increased root length, activity, wet weight, D/L-CD enzyme activities, endogenous H_2_S concentration, as well as CAT, SOD, and POD activities. These results suggest that NaHS treatment not only enhances plant acclimation to various stresses but also improves overall plant health.

After treatment with NaHS, a significant number of DEGs (970 up-regulated and 2175 down-regulated) were identified in the roots of the experimental group. GO enrichment analysis revealed that these DEGs are associated with large-scale biological processes and molecular functions, with cellular processes and metabolic processes being the predominant components of gene enrichment. DEGs were mainly enriched in components related to cells, organelles, catalytic activity and metabolism, indicating that DEGs are mainly concentrated in secondary metabolic processes, which is comparable to previous research results [54–57]. Additionally, these DEGs were mapped to KEGG pathways, highlighting significant differences in phenylpropanoid biosynthesis, photosynthesis, linolenic acid metabolism, and tyrosine metabolism pathways (Fig. 5c), which aligns with previous studies on biological processes [6, 26, 30]. Our study uniquely demonstrates that treating tobacco seedlings with NaHS aims to enhance their immunity rather than solely responding to various biological stresses.

TFs are known to play important roles in the developmental responses of plant roots. Some of the key TFs involved include MYB, EFR (EF-TU RECEPTOR), bZIP (basic region/leucine zipper motif), GRF, and MCM1/AGAMOUS/DEFICIENS/SRF (MADS)-box. Various TFs, such as GRF, MYB, and GATA, have been identified as important for root development. It is suggested that NaHS-treated TFs may be significant in the context of tobacco root development. Notably, several TFs directly regulate tobacco root development in distinct ways, although the interplay between these factors remains to be fully explored. The transcriptome sequencing data presented in this study offer insights into the regulatory networks and novel regulatory factors associated with enhancing NaHS treatment (Fig. 6). Particularly, the GRF transcription factor is highlighted for its unique role in plants, where it is known to regulate plant cell size, chloroplast proliferation, pistil development, and various growth and developmental processes, including responses to osmotic stress [58, 59]. GRF TFs are characterized by the presence of two conserved domains, QLQ (Gln, Leu, Gln) and WRC (Trp, Arg, Cys) [58]. The miR396-GRF regulatory module has been identified as a key player in regulating root development, with studies showing its significant effect on the size of the meristem in *Arabidopsis* [60, 61]. Overaccumulation of miR396 can disrupt the longitudinal structure of roots, as this microRNA is responsible for controlling the transition between stem cells and transit enhancing cells in *Arabidopsis*. Additionally, the MYB transcription factor family in plants, known for its diverse functions, plays a crucial role in regulating various physiological processes including plant development, metabolism, and responses to biotic and abiotic stresses [62, 63]. This highlights the intricate interplay of different TFs in orchestrating plant growth and development. Furthermore, under NaHS treatment, the up-regulation of MYB, GRF, and GATA genes suggests their close involvement in the physiological response to NaHS treatment, promoting root development and enhancing defense mechanisms against biological and abiotic stresses.

Metabolomics is a powerful tool for identifying and quantifying small-molecule metabolites within biological cells, enabling researchers to investigate the accumulation patterns of these metabolites [26]. This technology has also been instrumental in uncovering changes in genes and metabolites across various tissues. Metabolites, which serve as both intermediate and end products, play a crucial role in regulating plant growth and development [22]. With plants containing a vast array of up to 200,000 metabolites, they serve as an excellent model for studying biosynthetic regulation [25]. Recent researches have delved into the genetic mechanisms underlying changes in plant metabolites [23, 30]. By employing a comprehensive targeted metabolomics approach, we successfully identified and annotated 384 metabolites in the MS2 database. These metabolites were found to be related to key KEGG categories such as ‘amino acid metabolism’, ‘carbohydrate metabolism’, ‘glycosylphosphatidylinositol (GPI) anchored biosynthesis’, and ‘plant hormone signal transduction’. Notably, our results are consistent with previous studies investigating biological processes underlying plant resistance [26, 27, 30, 55].

The comparative analysis of metabolomics and transcriptome is valuable in elucidating the biological processes and mechanisms involved in regulatory responses. In plant roots, the regulation of H_2_S is generally dependent on concentration. Studies have shown that as the NaHS concentration increases, the length and number of cucumber roots initially increase and then decrease [30], mirroring the trend observed in the length and density of lateral roots in tomatoes [10]. Additionally, the regulation of H_2_S in tomato lateral root growth is related to H_2_O_2_ and methane signaling pathways [64, 65]. Notably, during NaHS treatment, plants activate a significant number of defense factors, particularly metabolites. The biosynthesis of BL, essential for various stages of plant development, emerged as a key enrichment pathway between CK and NaHS-treated groups. The BRI1 receptor receives BR signals on the cell surface through two mechanisms to activate BRI1 activity. One mechanism involves BAK1 as a co-receptor that interacts with BRI1 in vivo and in vitro. Upon BR stimulation, BRI1 and BAK1 form heterodimers and undergo mutual phosphorylation [66–69], thereby regulating root cell development. The other mechanism entails BR binding to the BRI1 homodimer, inducing a conformational change that activates its activity and subsequently phosphorylates BAK1 [70]. Phosphorylated BRI1 and BAK1 then act on downstream components BES1 and BZR1 in the nucleus to receive upstream signals, ultimately regulating root cell division or differentiation [70–73]. This study observed a significant up-regulation of the metabolite BL after NaHS treatment, but the role of H_2_S in regulating BR-induced root development remains unclear. Heyman et al. demonstrated that BR can enhance QC cell division by inducing ERF115 expression and influencing PSK5 expression [74]. The up-regulation of the ERF115 gene in this study promoted QC cell division and stimulated tobacco growth.

Auxin can induce PLT expression, however, BZR1-mediated BR signaling can inhibit its expression, indicating that BR and auxin have opposing effects on QC cell division [70]. This interaction may be a key factor in NaHS regulation of BR-induced root development. Recent research has demonstrated that BR signaling can suppress SHY2 expression by promoting BES1 expression, with BES1 also directly influencing PINFORMED 7 (PIN7) to modulate root meristem development [75]. The above results indicate the intricate interaction between BR signaling pathway TFs and auxin regulates root stem cell niche, root apical meristem, root cell growth and lateral root formation. After NaHS treatment, increasing BR levels and reducing auxin levels may enhance root development. Furthermore, the down-regulation of xyloglucan endotransglucosylase encoded by the TCH4 gene suggests de novo incorporation of xyloglucan into the cell wall and plant morphogenesis [76, 77]. Consequently, the NaHS-mediated regulation of BR-induced TCH4 gene down-regulation could affect cell wall relaxation. Further studies are needed to elucidate the specific downstream target genes of BR in the NaHS response.

Amino acids are essential for protein synthesis. Previous studies have shown that amino acids, as intermediate or final metabolites of certain metabolic pathways, are also involved in the regulation of multiple metabolic pathways. These metabolic pathways are related to other physiological and biochemical metabolic pathways in plants and affect many physiological processes of plants [26]. In this study, the concentrations of aspartate, lysine, threonine, methionine, and isoleucine after NaHS treatment were significantly higher than those in CK. The aspartate family pathway includes four major essential amino acids (EAA): lysine, methionine, threonine, and isoleucine, and is also closely related to homoserine, glutamic acid, glycine, and proline.

After NaHS treatment, plants modify specific amino acids by changing the amino acid metabolism pathways, thereby maintaining the stability of functional proteins and metabolic enzymes. Judging from the source of the synthetic carbon skeleton, the amino constitute plant proteins and enzymes can be divided into five categories: 3-phosphoglycerate, α-ketoglutarate, pyruvate, oxaloacetate, and aromatic amino acids from the source of the synthetic carbon framework [49]. Collective analysis results showed that the application of H_2_S significantly changed the contents of several amino acids in tobacco roots (Fig. 15). After NaHS treatment, various amino acids were found in tobacco roots. Alpha-ketoglutarate-arginine content was significantly increased. The content of asparagine in α-ketoglutarate group, 3-phosphoglycerate group, serine and aromatic tyrosine groups was significantly reduced. Arginine is an amino acid with the highest nitrogen-to-carbon ratio. It is not only protein component but also a precursor for the biosynthesis of nitric oxide, polyamines and proline [50]. The large amount of arginine accumulated in tobacco roots can make the development of tobacco roots more vigorous and effectively promote the growth and development of tobacco.

NaHS treatment can positively and negatively regulate tobacco amino acid metabolism through multiple pathways (Fig. 13). NaHS treatment significantly reduced the content of *AnsA* gene and asparagine content in tobacco roots. The reason may be that hydrogen sulfide promotes the deamination of asparagine to aspartate, which is converted into glutamate and glutamine through the tricarboxylic acid cycle [78]. After NaHS treatment, the content of α-ketoglutarate family arginine and aromatic tyrosine in tobacco roots significantly increased, while the content of 3-phosphoglycerate family serine significantly decreased. Arginine and aspartate are important intermediates in the urea cycle. NaHS treatment can promote the conversion of aspartate into arginine by accelerating the urea cycle, thereby significantly increasing the arginine content.

Arginine can regulate plant growth and development of plants through its metabolites and related enzymes (Fig. 14c). For example, arginine produces NO under the catalysis of NOS, and regulates root growth and differentiation, induces flowering, and disrupts dormancy and gene expression through NO [75, 79, 80]. In addition, arginine can also be catalyzed by enzymes such as arginine decarboxylase (ADC) to form polyamine (PA), which can induce the formation of lateral roots, regulate fruit development, and promote flower bud differentiation and pollen germination through PA [81]. The synthesis of aromatic amino acids requires phosphoenolpyruvate in the glycolytic pathway and erythritol-4-phosphate produced in pentose phosphate pathway as substrates, while shikimate is synthesized through the shikimate pathway. The synthesized chlorate is then converted into tryptophan, phenylalanine and tyrosine [82]. Tyrosine content in tobacco increased significantly after NaHS treatment, suggesting that it may be related to the coordinated regulation of the pentose phosphate pathway, glycolysis pathway and shikimate pathway. The reason may be that H_2_S can increase the expression of enzyme proteins related to the bile acid synthesis pathway in plants [83, 84]. However, the coordinated regulation mechanism of H_2_S on plant carbon and nitrogen metabolism is relatively complex, and it is still necessary to use molecular biology methods to analyze the plant nitrogen metabolism mechanism.

This study provides valuable molecular information and new genetic engineering clues for further research on promoting plant growth. However, the functions of most of the DEGs or DAMs identified in this study remain largely unknown, and we will continue to investigate the functions of these genes and metabolites.

## Conclusions

In summary, differences between CK and NaHS treatment groups were analyzed through physiology, transcriptomics and metabolomics. Results showed that NaHS promoted root growth of tobacco seedlings and made tobacco seedlings resistant to pests and abiotic stresses. We discussed possible mechanisms between gene expression and metabolite biosynthesis. Brassinosteroid biosynthesis and aspartate metabolism may play important roles in promoting root development of tobacco seedlings. The key genes for brassinosteroid biosynthesis and regulatory genes for aspartate metabolism and their promoting effects on root development need to be further studied and verified.

This study offers valuable insights into the promotion of tobacco root growth, which is significant for enhancing tobacco cultivation practices. The findings suggest potential opportunities for tobacco farmers to minimize pesticide usage and safeguard soil health.

## Materials and methods

### Plant materials, growing conditions and treatments with NaHS and other chemicals

Tobacco seeds of the Yunyan 87 (*Nicotiana tabacum* cv. Yunyan 87) were provided by Wuhan Tobacco Research Institute, Hubei Province. Healthy seeds were surface sterilized alternately with 70% (v/v) ethanol solution for 10 min, and 10% (v/v) sodium hypochlorite solution for 5 min, and then washed repeatedly with distilled water. Seeds were then sown on Murashige and Skoog (MS) medium and stored in the dark at 26 ± 1 °C, a 16/8 h day/night cycle, and 65–70% relative humidity.

The seedlings were grown in the trays until they developed their fourth leaf. Then these intact four-leaf seedlings were carefully removed from the trays and washed their roots with running water. Seedlings of the same size were selected and acclimated in tap water for 24 h, and then treated with different concentrations of NaHS (0, 200 mg/L, 400 mg/L, 600 mg/L, 800 mg/L). In consideration of the water requirements of tobacco seedlings and the air-drying conditions in the greenhouse, each seedling was treated with 300 mL of NaHS solution each time. The NaHS-treated solutions were replaced every 2.5 days for 10 consecutive days. Normal deionized water was used as a control (CK). The experiment was conducted under natural conditions in the greenhouse of Hubei University. The daytime temperature is 25 °C (14 h) and the night temperature is 22 °C (10 h).

In each omics analysis, tobacco seedlings were treated with CK and NaHS for 10 days, and tobacco roots were collected from 3 plants (*n* = 3), frozen in liquid nitrogen immediately after treatment, and stored at -80 °C until use.

### Measurement of growth and root activity

After 10 days of treatment, directly measure the length of the plant root system with a ruler. Growth and other morphological characteristics were determined by fresh weight. Tobacco plants were photographed after 10 days after treatment. For this purpose, treated and untreated seedlings were collected and their fresh weight was determined using a digital scale.

To measure root activity, we used the 2,3,5-triphenyl tetrazolium chloride (TTC) method [85, 86]. Briefly, 0.5 g of root sample was added to an equal volume of 10 mL of phosphate buffer and 0.4% TTC, mixed in a test tube, and incubated in the dark at 37 °C for 2 h until the root tip turned red color again. The reaction was then terminated with 2 mL of 1 mol/L sulfuric acid. Next, we cut off the red root tip, completely immersed it in 10 mL of methanol in a sealed graduated test tube, and incubated it at a temperature of 30–40 °C until the root tip completely turned white. Finally, the OD value of the above extract was measured using spectrophotometry (SP-756PC) at 485 nm. Blind testing can be used as a reference. All experiments were performed in triplicate. Root activity was calculated using the formula:

$$\:Root\:activity\hspace{0.17em}=C/(W\times T)$$

where C is the degree of tetrazole reduction (µg), W is the root weight (g), and T is the time (h).

### Determination of the endogenous H_2_S contents and the activities of L/D-CD

We determined endogenous H_2_S levels as previously described [13]. However, the protocol has been slightly modified. Initially, about 0.1 g of tobacco roots were ground in 0.9 mL of 20 mmol/L Tris-HCl (pH 8.0) and centrifuged to separate the components. The supernatant was carefully collected into a vial, followed by the addition of 1% Zn(AC)_2_, and finally sealed with a rubber stopper. The H_2_S released during the reaction was absorbed by Zn(AC)_2_. After reacting at 37 °C for 40 min, we added 100 µL of 20 mmol/L DPD (7.2 mol/L HCl) and 100 µL of 30 mmol/L FeCl_3_ (1.2 mol/L HCl) and left it react for 5 min. Finally, the absorbance value at 670 nm were measured, and a standard curve was drawn based on the Na_2_S concentration gradient. All experiments were performed in triplicate, and final data were expressed as the mean ± standard error of three replicate measurements.

 The activity of the L/D-CD enzyme was determined by measuring the amount of H_2_S released per minute during the degradation of L/D-Cys by L/D-CD enzyme, as described previously with some modifications [87]. First, we ground 0.1 gram of tobacco root in a mortar containing 0.9 mL of 20 mmol/L Tris-HCl (pH 8.0) and centrifuged. 100 µL of the resulting supernatant was added to 100 µL of 0.8 mmol/L L/D-cysteine, 400 µL of 2.5 mmol/L DTT, and 400 µL of 100 mmol/L Tris-HCl. The pH was set to 9 for LCD activity measurement and 8 for DCD activity measurement. The H_2_S content is detailed in Sect. 1. After incubation at 37 °C for 40 min, 100 µL of 20 mmol/L DPD in 7.2 mol/L HCl was added to 100 µL of 30 mmol/L FeCl_3_ in 1.2 mol/L HCl. Finally, absorbance at 670 nm was recorded after 5 min.

### Determination of the enzymatic activity of antioxidant enzymes and analysis of MDA content

CAT activity was determined by monitoring the decomposition of H_2_O_2_ at 240 nm for 3 min with a slight modification [88, 89]. The reaction mixture consisted of 2 mL of 50 mmol/L sodium phosphate buffer (pH 7.0), 1 mL of 0.2% H_2_O_2_, and 0.2 mL of enzyme extract. The optical density (OD) at 240 nm was measured with a spectrophotometer after 45 s and 60 s upon adding the extract to the cuvettes. The difference in OD between the 45-second and 60-second readings was used to calculate CAT activity. One unit (U) was defined as the amount of enzyme that caused a change in absorbance of 0.01 per minute.

The POD activity was assessed as described previously [26, 90]. Briefly, 5.0 gram of washed tobacco roots were treated with 0.0, 200, 400, 600, and 800 mg/L NaHS for 10 days, then weighed and stored in 4 mL of 50 mM potassium phosphate buffer (pH 7.6) at 4 °C. These roots were homogenized with a mortar and pestle. We transferred the homogenate to a test tube and centrifuged at 3000 g for 10 min. Then we transferred the supernatant to a 25 mL volumetric flask. Next, we extracted the pellet twice with 5 mL of phosphate buffer and added the supernatant to the volumetric flask. The volume was attached to the balance and stored at a low temperature. For the enzyme activity assay, 0.1 mL of the enzyme extract was added to 2.9 mL of 0.05 mol/L phosphate buffer, along with 1.0 mL of 2% H_2_O_2_ and 0.05 mol/L guaiacol. The mixture was then incubated in a water bath at 37 °C for 15 min, following the protocol. As a control, the enzyme extract was boiled for 5 min. Next, the reaction system was transferred to an ice bath. The reaction was stopped with 2.0 mL of 20% trichloroacetic acid, filtered or centrifuged at 5000 g for 10 min. Enzyme activity was determined by incubating the enzyme extract with a mixture of H_2_O_2_ and guaiacol, followed by measuring absorbance at 470 nm. The formula used to calculate POD activity was:

$$\mathrm{POD}\;\mathrm{Activity}=\;\frac{\mathrm\Delta\;{\mathrm A}_{470\times}{\mathrm V}_{\mathrm t}}{\mathrm W\times\mathrm{Vs}\times0.01\times\mathrm t}$$

where ΔA_470_ is the change in absorption, W is the weight of tobacco roots (g), t is the time of reaction (minute), Vt is the total volume of enzyme extract (mL), and Vs is the volume of test enzyme extract. One unit (U) of POD activity is equivalent to a 0.01 change in A_470_ per minute.

The SOD activity described in a previous study [88] was determined using the following method: 0.5 gram of fresh tobacco roots were treated with NaHS at concentrations of 0.0, 200, 400, 600 and 800 mg/L for 10 days. These roots were then homogenized with 5 mL of prechilled phosphate buffer in a mortar on an ice bath. The homogenate was filtered through gauze, centrifuged at 1000 g for 20 min, and finally the supernatant was taken as crude enzyme extract. All steps were performed at 0–4 °C. The SOD activity was measured as follows: we added 0.05 mL of enzymatic extract to 0.05 mol/L potassium phosphate buffer (pH 7.8), and added 0.3 mL of 130 mmol/L methionine, 750 µmol/L nitro blue tetrazolium chloride (NBT), 100 µmol/L EDTA-Na2 and 0.32 mL of 20 µmol/L riboflavin. The reaction mixture was incubated in the chamber under 4000 LX fluorescent light for 20 min. The reaction was started by turning on the fluorescent lamp and ended after 5 min by turning off the fluorescent lamp. The increase in absorbance at 560 nm was measured using the NBT photoreduction method. The reaction mixture without enzyme extract was used as a control and incubated in the dark. A control reaction mixture without enzyme extract was incubated in the dark. One unit of SOD was defined as the amount of enzyme necessary to inhibit the photoreduction of NBT by 50% compared to a reaction mixture without plant extract. The SOD activity was calculated using the formula:

$$\mathrm{Total}\;\mathrm{SOD}\;\mathrm{activity}\;=\frac{\;\left({\mathrm A}_{\mathrm{CK}-}{\mathrm A}_{\mathrm E}\right)}{0.5\times{\mathrm A}_{\mathrm{CK}}\times\mathrm W\times{\mathrm V}_{\mathrm t}}$$

$$\mathrm{SOD}\;\mathrm{activity}\;=\;\mathrm{Total}\;\mathrm{SOD}\;\mathrm{activity}/\mathrm{protein}\;\mathrm{content}$$

where A_CK_ and A_E_ are the absorbances of the control and sample tubes, V is the total volume of the sample tube, Vt is the volume of the test sample, and W is the weight of the fresh sample.

To determine MDA content, we used the thiobarbituric acid reactive substances (TBARS) method [26, 91]. 1 gram of the treated tobacco roots was weighed and then mixed with small amount of quartz sand and 2 mL of 10% trichloroacetic acid. The mixture was ground for homogenization, followed by the addition of 8 mL of 10% trichloroacetic acid for further grinding. The homogenate was centrifuged at 4000 rpm for 10 min, and the supernatant was malondialdehyde extract. 2 mL of extract was added to each clean test tube, followed by 2 mL of distilled water in the control tube, and then 2 mL of 0.6% thiobarbituric acid solution was added to each tube. The mixture was shaken well, allowed to react in a boiling water bath for 15 min, rapidly cooled, and then centrifuged. The absorbance values (A) of the supernatant were measured at 532 nm, 600 nm and 450 nm, respectively, with three replicates for each treatment. The MDA content was calculated using the following formula:

$$\mathrm{MDA}\;\mathrm{content}\;=\;\left[6.45\times\left({\mathrm A}_{532-}{\mathrm A}_{600}\right)-0.56\times{\mathrm A}_{450}\right]\times\;{\mathrm V}_{\mathrm T}/\left({\mathrm V}_0\times\mathrm W\right)$$

where A_532_, A_600_, and A_450_ represent the absorptions of the sample tubes at 532 nm, 600 nm, and 450 nm, respectively. V_T_ denotes the total volume of the extract in mL, V_0_ is the measured liquid volume in mL, and W stands for the fresh weight of the tobacco tissue in grams.

### Transcriptomics analysis

Tobacco seedlings were collected for hydroponic experiments. After being treated with 600 mg/L NaHS for 10 days, these roots were frozen in liquid nitrogen and sent to Majorbio Bio-Pharm Technology Co. Ltd. (Shanghai, China) for transcriptome sequencing, with three biological replicates for each treatment. During sequencing, RNA was extracted from tobacco roots. After filtering the raw data, the sequencing error rate and GC content distribution were checked to obtain clean measurement results for subsequent analysis. FPKM values were used to indicate transcript or gene expression levels. The specific steps of data processing were carried out as described by Meng et al. [88, 89].

DEGs between the two groups were evaluated using the DESeq2 software with criteria of |log2Fold Change| ≥ 1 and FDR < 0.05 [88, 89]. Hypergeometric distribution testing was performed for KEGG pathways and GO terms. A heatmap was generated to illustrate the bidirectional hierarchical grouping of the identified DEGs and samples. ToGO was used to conduct GO analysis of BP, MF, and CC to elucidate the relationship between DEGs and plant development-related pathways.

GO pathway analysis encompassing BP, MF, and CC was conducted using TopGO to elucidate the relationships between DEGs and pathways associated with plant development.

### Metabolomics analysis

Tobacco seedlings were collected for hydroponic experiments. After treatment with 600 mg/L NaHS for 10 days, the roots were frozen in liquid nitrogen. Sample preparation and data analysis for metabolic analysis were performed at Majorbio Bio-Pharm Technology Co. Ltd. (Shanghai, China) following standard procedures, and six biological replicates of each treatment were analyzed. Metabolite extraction from frozen roots using 400 µL of methanol/water (4:1) solution. The ground materials were processed with a high-throughput tissue grinder at -20 °C and 50 Hz for 6 min, then sonicated at 4 °C and 40 kHz for 30 min, and finally centrifuged at 4 °C and 13,000 g for 15 min. The obtained supernatant was carefully transferred to a sample vial for LC-MS/MS analysis. A pooled quality control (QC) sample was created by combining equal volumes of all individual samples and analyzed using the same method as the analytical samples.

### Quantitative fluorescence PCR analysis

Quantitative fluorescence PCR was used to detect the expression levels of twelve differentially expressed unigene transcripts and sequenced using the same RNA samples. Specific primers were designed using Primer Premier 6.0 and were listed in Table S18. The housekeeping gene used in this study was ubiquitin. The first cDNA strand fragments were synthesized from total RNA using the PrimeScript™ RT Master Mix Kit (Takara, Japan) according to the user manual. qRT-PCR was performed on an ABI7500 real-time fluorescence quantitative PCR machine (Applied Biosystems, Foster City, USA).

The housekeeping gene used was ubiquitin. Following the user manual instructions, initial cDNA strand fragments were synthesized from total RNA with the PrimeScript™ RT Master Mix Kit (Takara, Japan). Subsequently, qRT-PCR was conducted on an ABI7500 real-time fluorescence quantitative PCR machine (Applied Biosystems, Foster City, USA), with three replicates for each sample. Each sample was tested in triplicate. The relative expression levels of the candidate genes were determined using the comparative threshold cycle method (2^−ΔΔCT^) [1, 40].

### Statistical analysis

All data analysis and graphics were performed using R version 4.1.2. Statistical significance was determined by Student’s t-test. Gene expression values in FPKM were normalized to Z-scores for generating the transcriptome and metabolome heatmaps. Graphs and heatmaps were created using the ggplot and heatmap packages in R. Each experiment was repeated three times.

### Supplementary Information

Supplementary Material 1.

Supplementary Material 2.

Supplementary Material 3.

Supplementary Material 4.

Supplementary Material 5.

Supplementary Material 6.

Supplementary Material 7.

Supplementary Material 8.

Supplementary Material 9.

Supplementary Material 10.

Supplementary Material 11.

Supplementary Material 12.

Supplementary Material 13.

Supplementary Material 14.

Supplementary Material 15.

Supplementary Material 16.

Supplementary Material 17.

Supplementary Material 18.

Supplementary Material 19.

Supplementary Material 20.

## Data Availability

RNA-seq data that support the findings of this study have been deposited in the Sequence Read Archive (SRA) at NCBI with the primary accession code PRJNA1103126.
