# Supplementary figures and images for: Metabolomics and transcriptomics combined with physiology reveal key metabolic pathway responses in tobacco roots exposed to NaHS
Source: BMC Plant Biol. 2024 Jul 18;24:680. doi: 10.1186/s12870-024-05402-z (PMC11256483; doi:10.1186/s12870-024-05402-z)

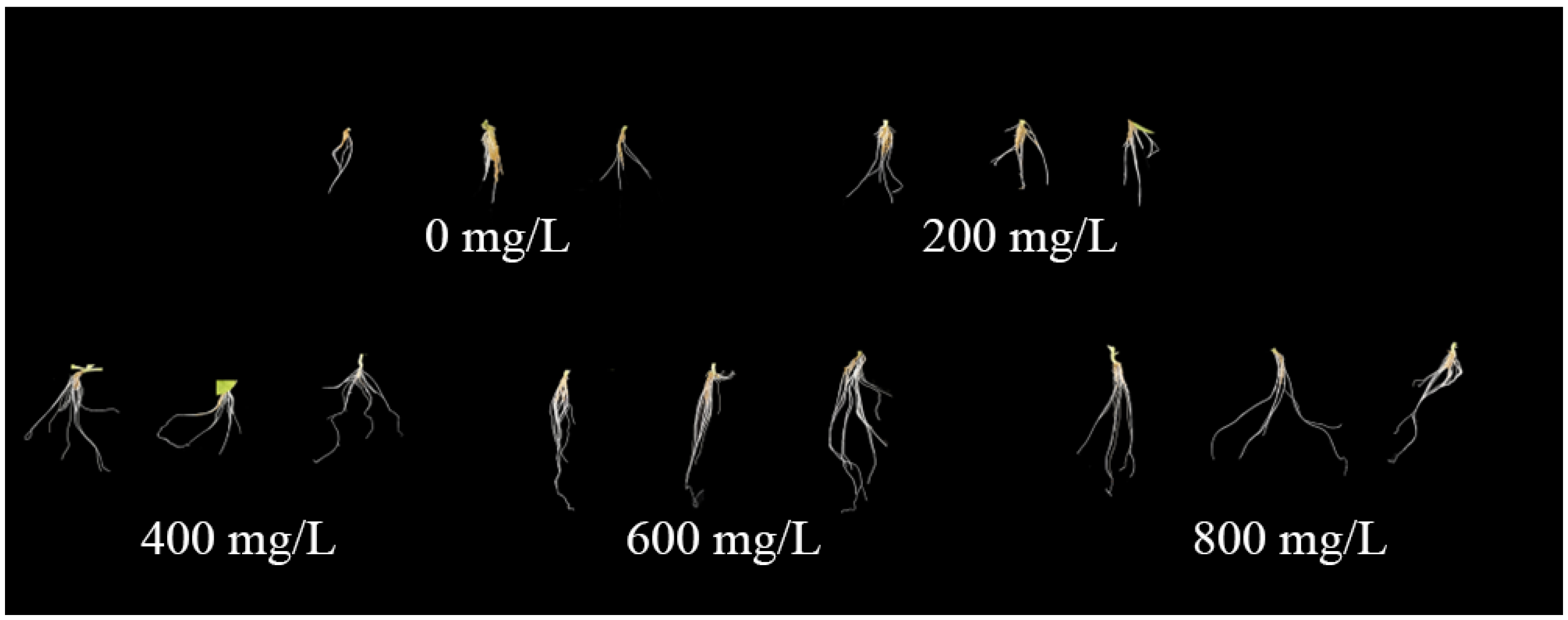

Supplement: Supplementary file 1 — Supplementary Material 1. [file 12870_2024_5402_MOESM1_ESM.tif]
